# The Relationship Between Cancer and Functional and Structural Markers of Subclinical Atherosclerosis: A Systematic Review and Meta-Analysis

**DOI:** 10.3389/fcvm.2022.849538

**Published:** 2022-05-04

**Authors:** Yuhong Diao, Zhixing Liu, Li Chen, Weiping Zhang, Dandan Sun

**Affiliations:** Department of Ultrasound Medicine, The First Affiliated Hospital of Nanchang University, Nanchang, China

**Keywords:** cancer, intima-media thickness, pulse wave velocity, flow-mediated vasodilation, systematic review and meta-analysis

## Abstract

**Objectives:**

The relationship between cancer and subclinical atherosclerosis has always been the focus of people's attention. We conducted a systematic review and meta-analysis by evaluating the effects of cancer on functional and structural markers of subclinical atherosclerosis:intima-media thickness (IMT), pulse wave velocity (PWV), and flow-mediated vasodilation (FMD).

**Methods:**

A comprehensive and systematic literature search was conducted on the internet. Sensitivity analysis, publication bias, standard mean difference (SMD), corresponding 95% confidence interval (95% CI), and subgroup analysis were performed for all relevant research indicators in the retrieved literature.

**Results:**

Forty-six studies were included, including 3,729 cancer patients and 2,404 healthy controls. Cancer patients had significantly thicker IMT [SMD (95%CI) = 0.290 (0.069 to 0.511), *P* = 0.010] and higher PWV [SMD (95%CI) = 0.392 (0.136 to 0.647), *P* = 0.003] compared with healthy controls. There was no significant difference in FMD [SMD (95% CI) = −0.192 (−0.527 to 0.144), *P* > 0.05). After subgrouping by age, male proportion, and treatment, the analysis results of IMT ≥ 50 years old, PWV and FMD < 50 years old, male proportion ≥50%, chemotherapy group, IMT and PWV radiotherapy group, and PWV endocrine therapy group were statistically significant (*P* < 0.05). There were no significant differences in other subgroup analyses, overall sensitivity analysis, and publication bias (*p* < 0.05).

**Conclusions:**

Cancer may promote subclinical atherosclerosis, and change the functional and structural markers of subclinical atherosclerosis such as IMT and PWV. Early intervention and prevention should be pursued.

## Introduction

The latest global cancer burden data for 2020 released by the World Health Organization's International Agency for Research on Cancer (IARC) shows that there were 19.29 million new cancer cases and 9.96 million cancer deaths worldwide in 2020. Cancer has become a common disease with high mortality and morbidity worldwide (1, 2), but with the progress of cancer treatment, the 5-year survival rate of cancer patients is getting higher and higher, and cancer has now become a chronic disease (2). At present, many studies show that cancer patients die from cardiovascular diseases in addition to cancer recurrence (3–6). The incidence of atherosclerosis in cancer patients is very high (4), which may be related to the systemic inflammatory response and impaired vascular endothelial function caused by the mechanism of cancer cell infection and corresponding chemoradiotherapy. Cancer patients all need to suffer from this change whether caused by simple cancer infection or its treatment. However, early detection and timely intervention can avoid the occurrence of atherosclerosis and even cardiovascular accidents.

Intima-media thickness (IMT), flow-mediated vasodilation (FMD), and pulse wave conduction velocity (PWV) are considered markers of atherosclerosis and provide important information about artery function and structure (7). These three markers have been widely used to evaluate subclinical atherosclerosis in order to improve prognosis and timely individualized treatment. Some studies have shown increased IMT, PWV, and reduced FMD in cancer patients compared to healthy controls (8–11). Conversely, some studies have produced inconsistent results (12–14). Currently, it is still controversial that cancer causes subclinical atherosclerosis and increased risk of cardiovascular disease, therefore we conducted this meta-analysis to further determine the relationship between cancer and subclinical atherosclerosis.

Since the available results are controversial, we conducted this meta-analysis to further evaluate differences in IMT, PWV, and FMD caused by cancer and to clarify the association between cancer and subclinical atherosclerosis.

## Article Types

Systematic Review articles. Our systematic reviews conform to the reporting guideline PRISMA.

## Manuscript Formatting

### Materials and Methods

#### Publication Search

We searched the databases of PubMed, Web of Science, and Medline for articles concerning the relationship among the effects of cancer infection or related treatments on functional and structural markers of the vasculature (IMT, FMD, and PWV). The articles published between the earliest available dates to 5 August 2021 were applied. The key terms were as follows: “IMT” or “intima media thickness” and cancer or “flow-mediated dilatation” or “FMD” and cancer or “PWV” or “pulse wave velocity” and cancer.

#### Study Selection

**Figure 1:**
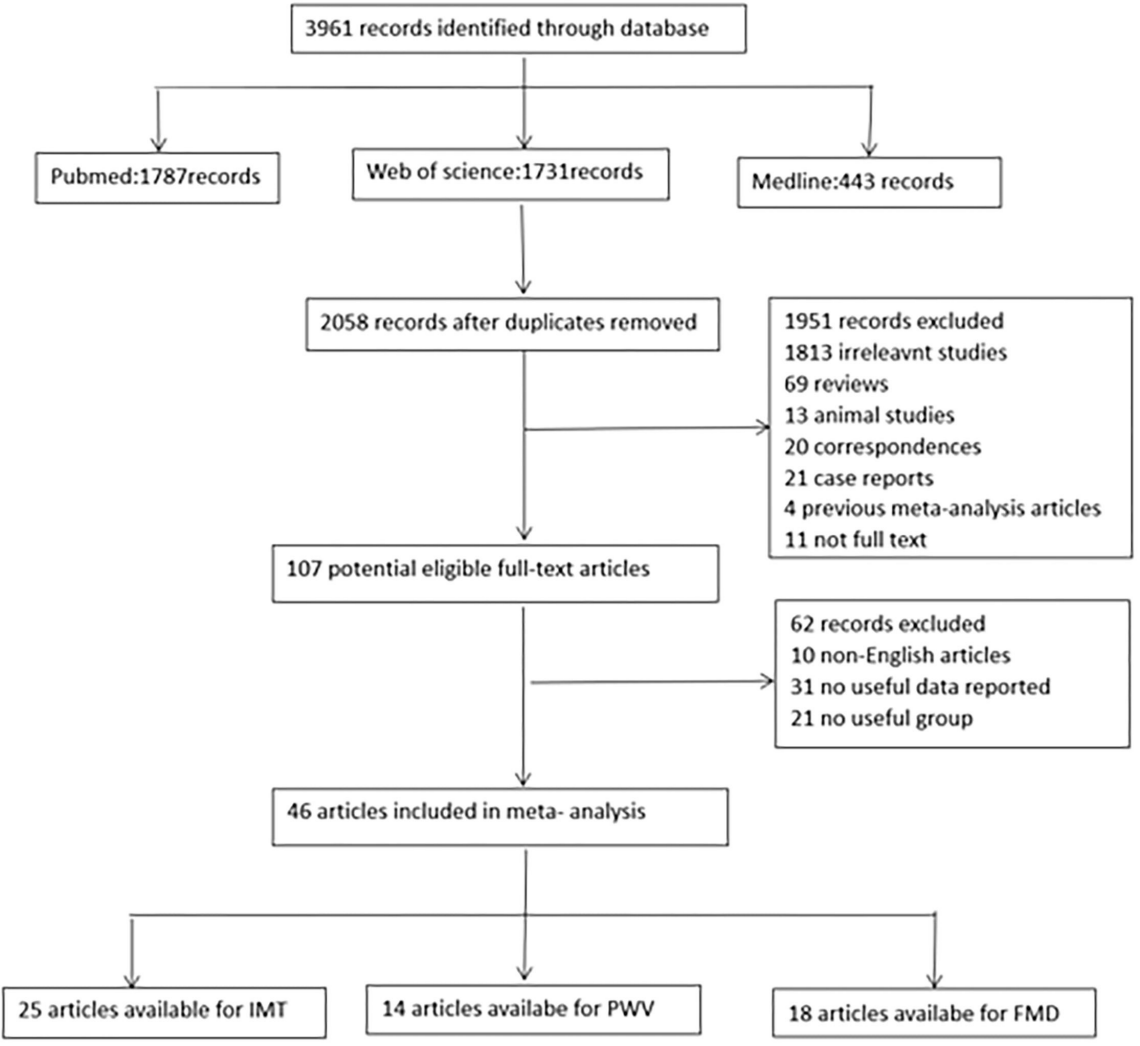
Flow chart for identification of studies. FMD indicates flow-mediated vasodilation; IMT, intima media thickness; PWV, pulse wave velocity.

Articles searched through the above retrieval methods were further screened using the following criteria. Figure 1 shows the filtering process of the studies.

##### Inclusion Criteria

1. The study should provide IMT, PWV, and/or FMD values.
2. The study should contain at least 2 independent groups or subgroups, 1 diagnosed with cancer and the other included healthy control subjects.

##### Exclusion Criteria

1. Find duplicate papers and keep one.
2. Take out reviews, case reports, letters, animal studies, and previous meta-analysis articles.
3. The study retrieved by abbreviations was not the markers of the vasculature.
4. No papers available in full.
5. The study was not published in English.

#### Data Extraction and Management

The data of the studies after the inclusion criteria and exclusion criteria were searched and extracted by two authors, describing in a standard format. In the screening process, they check each other out and come up with a consistent conclusion. The basic information to be collected is as follows: author, country, region, year, study design, site measured, sample size, mean age, the proportion of male, proportion of hypertension, proportion of smokers, proportion of patients with diabetes, the proportion of patients with hypertension, mean body mass index, mean systolic blood pressure, mean diastolic blood pressure, mean fasting blood glucose, mean total cholesterol, mean triglyceride, mean high-density lipoprotein, mean low-density lipoprotein, inflammatory markers, and the values of IMT, PWV, and FMD.

#### Data and Statistical Analysis

All of the study outcomes (values of IMT, PWV, and FMD) were transformed into mean and SD forms, which were performed using STATA 12.0 software (Stata Corp. College Station, TX, USA). Considering that the study values were measured on different sites, we calculated the standardized mean differences (SMDs) and 95% confidence intervals (95% CIs). Statistical heterogeneity between studies was assessed with the value of *I*^2^. If *I*^2^ was more than 50%, a fixed-effects model was considered (15). If *I*^2^ was no more than 50%, a random-effects model was applied. In the meantime, the sub-group analysis was used for potential confounders (mean age and male percentage). And univariate meta-regression analysis for the covariates (sample size, age, sex, region, and site measured) was also conducted. In addition, the sensitivity analysis was performed by removing studies one by one to assess the impact of each study on the overall outcome (16). Using funnel plots, the Begg model and Egger test evaluated qualitatively publication bias.

## Results

### Characteristics of Identified Studies

The search process is presented in the flow chart (Figure 1). In total, 3,961 articles were identified. According to the inclusion and exclusion criteria, 46 articles were finally applied in the meta-analysis, of which 25 could be used for IMT,14 studies included PWV, and 18 studies reported FMD values (8–14, 17–55) (Supplementary Materials Supplementary Tables S1–3). The articles were selected from different regions. Nine of the articles are from Asia, 22 are from Europe, 12 are from North America and two are from South America. They were published from 1991 to 2021. Their average age ranged from 9.5 to 70.6, and the proportion of men ranged from 0 to 100 percent. Their treatment methods are complex and varied, including radiotherapy, chemotherapy, endocrine therapy, and mixed treatment or early untreated treatment. In addition, different articles measured IMT, PWV, and FMD at different anatomical sites. More clinical characteristics included in the study, such as smokers, BMI, blood pressure, fasting glucose, and lipid ratio, are shown in Supplementary Table S4 of the online Supplementary Materials table.

#### The Relationship of Cancer and IMT

**Figure 2:**
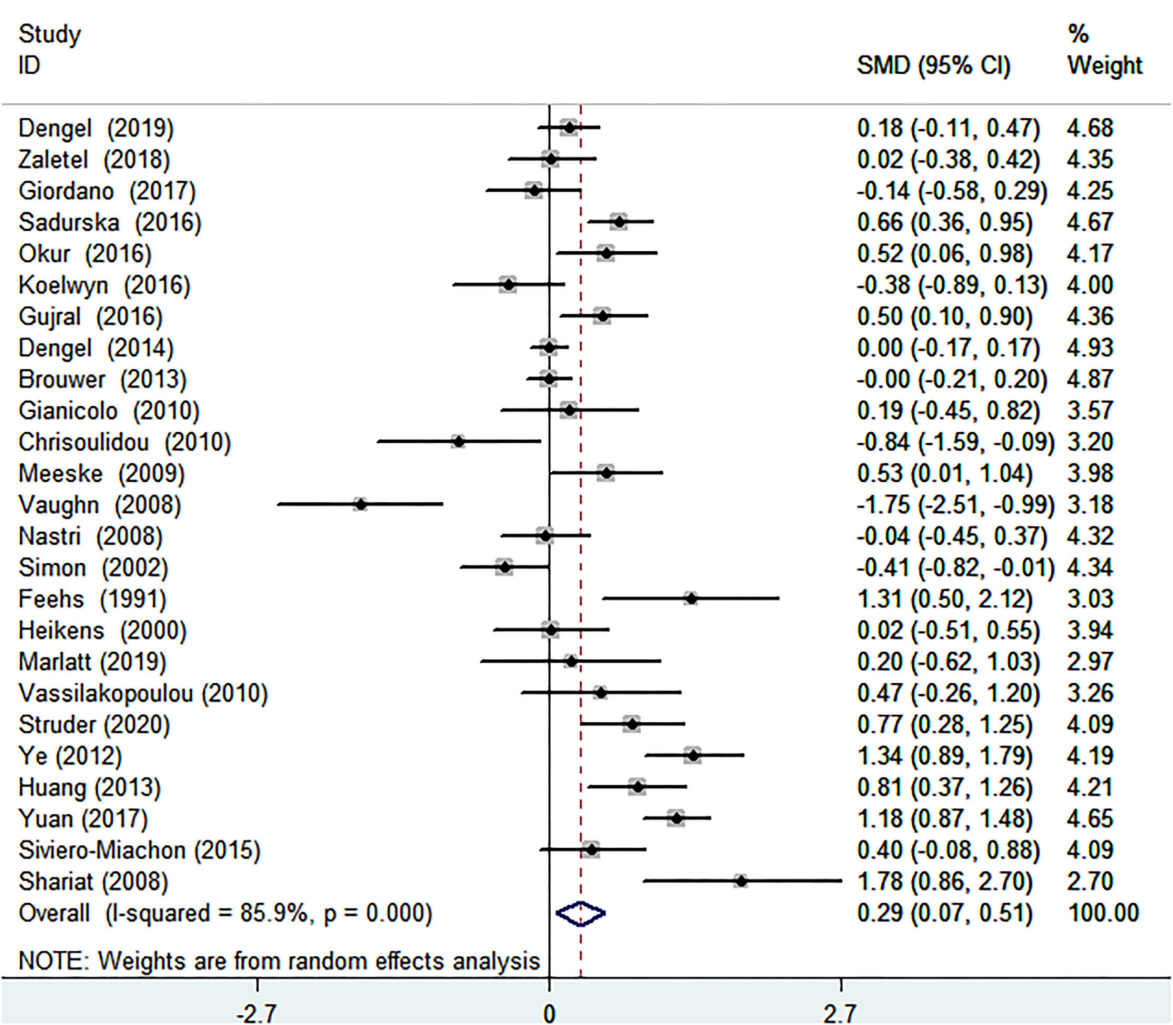
Forest plot of intima-media thickness in patients with cancer and controls without cancer. SMD, standard mean difference; CI, confidence interval.

After analyzing the 25 articles comprising 1,927 cancer patients and 1,077 healthy controls collected, we concluded that the IMT of patients with cancer was significantly increased than that of controls without cancer [SMD (95% CI) = 0.290 (0.069–0.511), *P* = 0.010] (Table 1; Figure 2). Because *I*^2^ = 85.9% and *P* < 0.001, which represented significant heterogeneity, the random-effects model was conducted in the meta-analysis. In terms of sub-group analyses, there was a thicker IMT in the following subgroups: age ≥50 years [SMD (95% CI = 0.451 (0.020, 0.883), *P* = 0.040], male ratio ≥ 50% [SMD (95% CI) = 0.451 (0.020, 0.883), *P* = 0.011], radiotherapy group [SMD (95% CI) = 0.713 (0.418, 1.008), *P* < 0.001], and chemotherapy group [SMD (95% CI) = 0.795(0.108, 1.481), *P* = 0.023] (Table 1). There was no significant change in the SMD (95%CI) by taking out studies one by one (Supplementary Materials Supplementary Table S5). Beyond that, the results of the Begg test (*P* = 0.834) and Egger test (*P* = 0.566) didn't show significant publication bias (Table 2). Analysis of subgroup, analysis of sensitivity, and meta-regression (Supplementary Materials Supplementary Table S8) failed to explain the source of heterogeneity.

**Table 1:** IMT, PWV, and FMD between patients with cancer and controls without cancer in overall and sub-group meta-analyses by age and female ratio.

**Variables**	**Group**	***N***	**SMD(95%CI)**	***p***	***I*^2^(%)**	**P_**hetero**_**
IMT						
All		25	0.290 (0.069 to 0.511)	**0.010**	85.9	<0.001
Age, years	<50	16	0.202 (−0.050 to 0.455)	0.116	84.0	<0.001
	≥50	9	0.451 (0.020 to 0.883)	**0.040**	87.5	<0.001
Male, %	<50	13	0.171 (−0.156 to 0.497)	0.306	85.1	<0.001
	≥50	12	0.417 (0.097 to 0.738)	**0.011**	87.5	<0.001
Therapy	Radiotherapy	12	0.713 (0.418 to 1.008)	**<0.001**	76.6	<0.001
	Chemotherapy	6	0.795 (0.108 to 1.481)	**0.023**	91.9	<0.001
	Endocrine therapy	3	−0.350 (−0.742 to 0.043)	0.081	47.6	0.149
	Other	4	0.018 (−0.112 to 0.148)	0.786	0	0.628
PWV						
All		14	0.392 (0.136 to 0.647)	**0.003**	77.0	<0.001
Age, years	<50	5	0.448 (0.195 to 0.702)	**0.001**	34.0	0.195
	≥50	9	0.346 (−0.035 to 0.727)	0.075	84.2	<0.001
Male, %	<50	7	0.406 (−0.075 to 0.888)	0.098	83.2	<0.001
	≥50	7	0.350 (0.088 to 0.612)	**0.009**	63.9	0.011
Therapy	Radiotherapy	3	0.704 (0.325 to 1.083)	**<0.001**	66.9	0.049
	Chemotherapy	5	0.463 (0.020 to 0.905)	**0.040**	73.0	0.005
	Endocrine therapy	1	1.081 (0.220 to 1.943)	**0.014**	-	-
	Other	5	0.068(−0.287 to 0.423)	0.706	64.8	0.023
FMD						
All		18	−0.192 (−0.527 to 0.144)	0.263	88.2	<0.001
Age, years	<50	9	−0.520 (−0.915 to −0.125)	**0.010**	89.4	<0.001
	≥50	9	0.214 (−0.444 to 0.872)	0.523	85.6	<0.001
Male, %	<50	13	0.099 (−0.374 to 0.572)	0.681	84.1	<0.001
	≥50	5	−0.797 (−1.351 to −0.242)	**0.005**	93.5	<0.001
Therapy	Radiotherapy	2	1.638 (−0.554 to 3.831)	0.143	90.4	0.001
	Chemotherapy	7	−0.853 (−1.682 to −0.023)	**0.044**	91.4	<0.001
	Endocrine therapy	3	−0.028 (−0.658 to 0.601)	0.930	67.3	0.047
	Other	6	−0.085 (−0.209 to 0.038)	0.176	0	0.997

*The results are in bold if p < 0.05*.

*N, number; SMD, standard mean difference; CI, confidence interval; Phetero, pvalue of heterogenicity; IMT, intima-media thickness; PWV, pulse wave velocity; FMD, flow-mediated vasodilation*.

**Table 2:** Publication bias evaluated by Begg's and Egger's tests.

	**Begg Test**		**Egger Test**	
	**Z Value**	***P*-Value**	***t* Value**	***P*-Value**
IMT	0.21	0.834	0.58	0.566
PWV	0.33	0.743	−0.69	0.501
FMD	0.00	1.000	−0.08	0.935

*IMT, intima-media thickness; PWV, pulse wave velocity; FMD, flow-mediated vasodilation*.

#### The Relationship of Cancer and PWV

**Figure 3:**
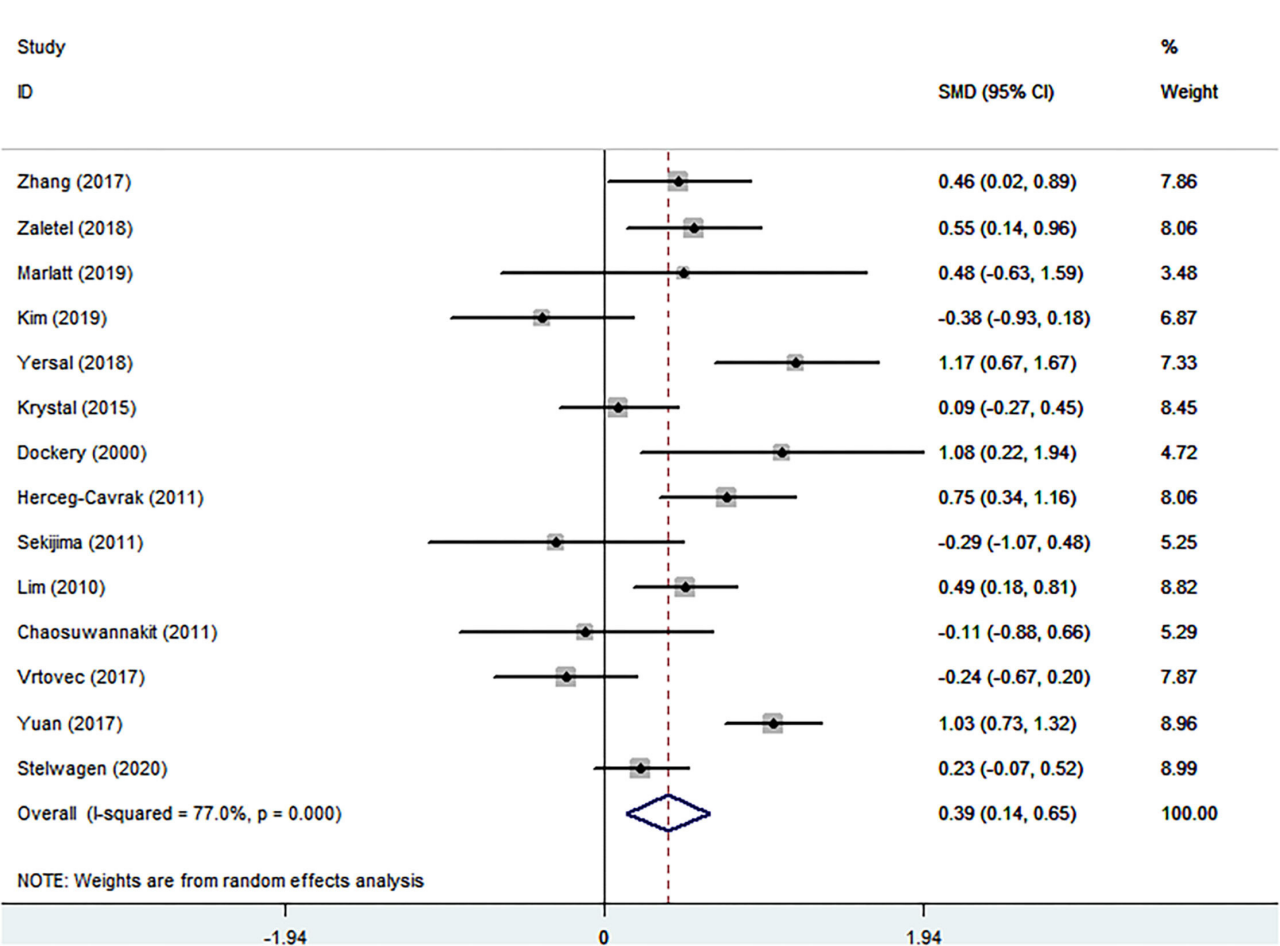
Forest plot of pulse wave velocity in patients with cancer and controls without cancer. SMD, standard mean difference; CI, confidence interval.

The conclusion that PWV was visibly increased in patients with cancer was suggested by comparing the summarized data of patients with cancer and healthy controls, SMD of 0.392 (95% CI = 0.136–0.647, *P* = 0.003) (Table 1; Figure 3). There was significant heterogeneity, *I*^2^ = 77.0%. In terms of sub-group analyses, there was a higher PWV values in the following subgroups: age <50 years [SMD (95% CI = 0.448 (0.195, 0.702), *p* = 0.001), male ratio ≥ 50% [SMD (95% CI) = 0.350 (0.088, 0.612), *P* = 0.009], radiotherapy group [SMD (95% CI) = 0.704 (0.325, 1.083), *P* < 0.001], chemotherapy group [SMD (95% CI) = 0.463 (0.020, 0.905), *P* = 0.040], and endocrine therapy group [SMD (95% CI) = 1.081 (0.220, 1.943), *P* = 0.014] (Table 1). There was no obvious publication bias from the results of the Begg test (*P* = 0.743) and Egger test (*P* = 0.501) (Table 2). There was no significant change in the SMD (95%CI) by taking out studies one by one (Supplementary Materials Supplementary Table S6). Moreover, analysis of subgroup, analysis of sensitivity, and meta-regression (Supplementary Materials Supplementary Table S8) failed to explain the source of heterogeneity.

#### The Relationship of Cancer and FMD

**Figure 4:**
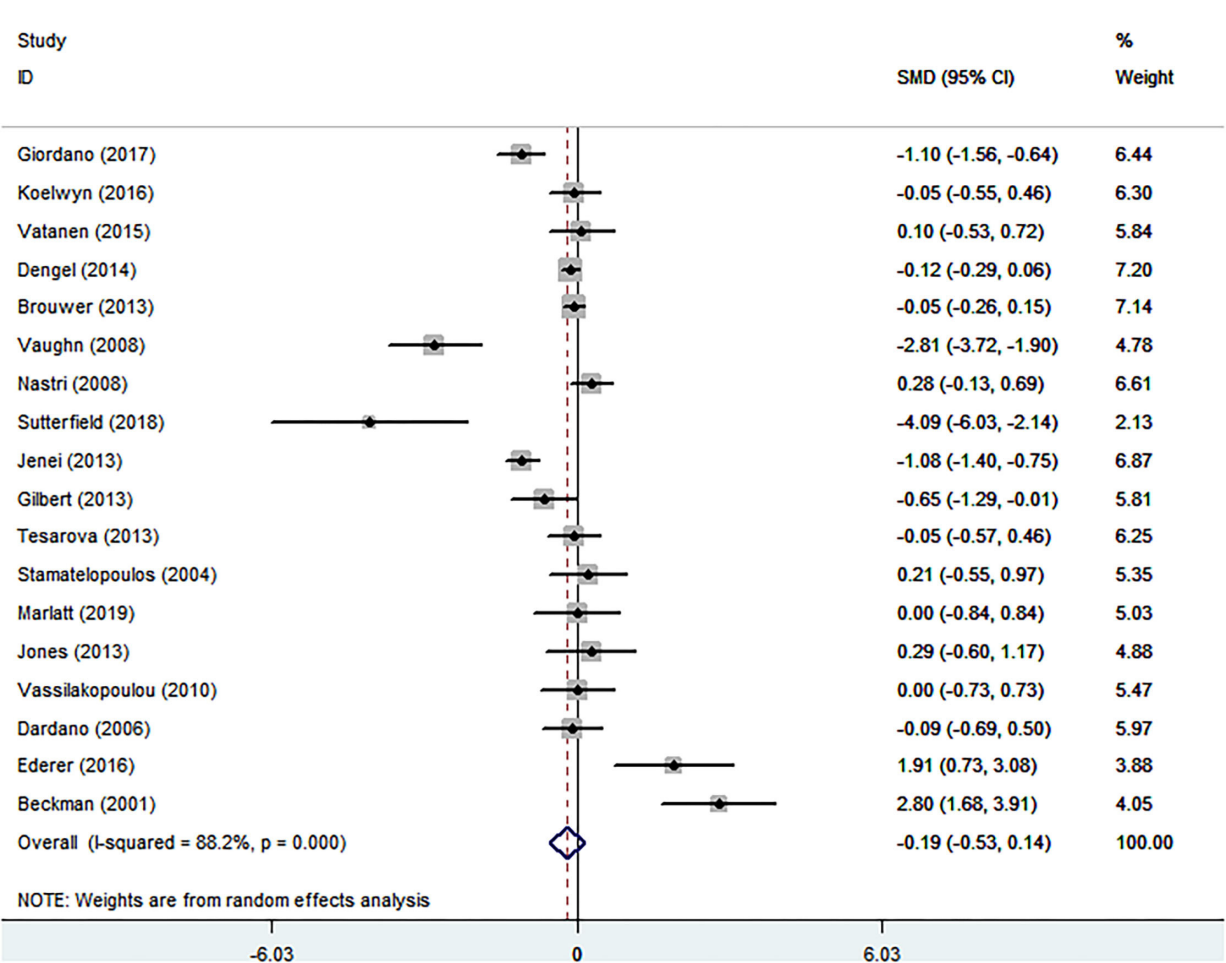
Forest plot of flow-mediated vasodilation in patients with cancer and controls without cancer. SMD, standard mean difference; CI, confidence interval.

Eighteen eligible studies were finally identified, involving 1,074 cancer patients and 672 healthy controls.The SMD of −0.192 (95% CI = −0.527 to 0.144, *P* = 0.263) was no significance (Table 1; Figure 4). The *I*^2^ statistic was 88.2%. In terms of sub-group analyses, there was a lower FMD in the following subgroups: age <50 years [SMD (95% CI) = −0.520 (−0.915, −0.125), *P* = 0.010], male ratio ≥ 50% [SMD (95% CI) = −0.797(−1.351, −0.242), *p* = 0.005], and chemotherapy group [SMD (95% CI) = −0.853 (−1.682, −0.023), *P* = 0.044] (Table 1). The Begg test (*P* = 0.743) and Egger test (*P* = 0.501) was no obvious publication bias (Table 2). There was no significant change in the SMD (95%CI) by taking out studies one by one (Supplementary Materials Supplementary Table S7). Analysis of subgroup, analysis of sensitivity, and meta-regression (Supplementary Materials Supplementary Table S8) failed to explain the cause of meaningless.

## Discussion

To provide evidence for the impact of cancer on arterial structure and function (IMT, PWV, and FMD) in relation to subclinical atherosclerosis, we pooled data from 46 full-text articles and conducted a meta-analysis of available data from 3,729 cancer patients and 2,404 healthy controls. The results showed that cancer patients had more severe impairment of vascular structure and function, with thicker IMT and higher PWV than healthy subjects. The carotid artery is generally selected for IMT measurement, which can be obtained by ultrasound examination. The new ultrasound technology–ultrafast pulse wave velocity–can accurately and quickly obtain the carotid PWV. Subgroup analysis was performed to exclude the effects of age and sex on vascular structure and function. In addition, by analyzing and comparing different treatment methods in subgroups, it can be concluded that chemotherapy has a significant effect on functional and structural markers of subclinical atherosclerosis, such as IMT, PWV, and FMD, leading to show increased IMT, PWV, and reduced FMD. Radiotherapy also has a significant effect on IMT and PWV, which can cause an increase in IMT and PWV. Due to the relatively small sample size of the endocrine therapy group, its efficacy needs to be further determined. According to the results of the meta-analysis, cancer can promote the progression of atherosclerosis, damage vascular structure, and function, and increase the occurrence of cardiovascular risk. So, early detection and timely treatment can greatly improve the patients' quality of life.

More cancer patients die from cardiovascular disease than from other causes (56). At present, subclinical atherosclerosis in tumor patients has always been the focus of attention, but the specific pathophysiological mechanism is still unclear. The progression of atherosclerosis is closely related to inflammatory response and immune dysregulation after cancer cell infection (57). Chronic persistent systemic inflammation can lead to loss of confluent luminal elastin layer and activation of glycoprotein in confluence lumen of the vascular wall, resulting in subcutaneous aggregation of low-density lipoprotein and increased circulating cholesterol in the body, thus triggering the expression of endothelial adhesion molecules and chemokine secretion, platelet-derived chemokine deposition and intimal immune cell infiltration (58–60). Besides, the multidirectional inflammatory factor MIF is closely related to LDL and can promote the occurrence and development of atherosclerosis. And many studies have confirmed the expression of MIF in various cancers (61–64).

Of course, antitumor drugs and chemoradiotherapy are also key causes of atherosclerosis. Radiotherapy is the key step leading to vascular injury. The vascular endothelium is sensitive to radiation, and its pathophysiological basis is direct damage to the vascular wall (65–68). Radiotherapy induces endothelial dysfunction, increases capillary permeability, activates inflammation, and leads to intimal hyperplasia, collagen formation and deposition, and fibrosis, which is conducive to the formation of atherosclerotic plaques (69). Testicular cancer patients who received chemotherapy were reported to have a 1.6 times higher risk of dying from cardiovascular disease 10 years after treatment than the general population (70). Cisplatin-based chemotherapy directly impairs vascular structure and function, increasing early cardiovascular events and vascular age at the time of treatment, but symptoms do not appear until many years have accumulated (71–74). Antimetabolites, antimicrobials, and tyrosine kinase inhibitors are chemotherapeutic agents with a high risk of atherosclerosis.

Cancer and atherosclerosis share common risk factors, such as age, smoking, a high-sugar, high-fat diet, and a sedentary lifestyle (75). Cancer patients often have metabolic abnormalities, especially glucose metabolism and lipoprotein abnormalities (76). The proliferation of cancer cells and the apoptosis of normal cells disrupt homeostasis. Dyshomeostasis leads to the development of atherosclerosis (77).

Men with cancer in our study were more likely to develop atherosclerosis than women. This may be related to higher levels of estrogen in women, which limits inflammation, increases nitric oxide production, and inhibits endothelial cell apoptosis (78). This finding is consistent with previous reports (79).

Our meta-analysis has several limitations. First, only English articles are selected for analysis. Secondly, the source of heterogeneity between studies has not been identified. Multivariate logistic regression on a larger sample is needed in the future to find the source of heterogeneity. Thirdly, some included studies lacked basic information, failed to conduct covariate analysis and adjustment for cardiovascular risk factors such as hypertension, diabetes, hyperlipidemia and smoking, failed to analyze the relationship between IMT, PWV, FMD and cholesterol level, failed to analyze inflammatory markers and other risk factors in cancer and health control databases, failed to show the association of the time point of the data was collected upon cancer diagnosis and treatment, and failed to judge whether the changes in IMT, PWV and FMD are cumulative responses over a long period of time after cancer treatment or short-term acute responses. Fourthly, the inclusion study excluded people who had already experienced a cardiovascular accident, and the cancer population in the study had a shorter follow-up period, so no association could be drawn between IMT, PWV, FMD and actual CAD event complications. Finally, the number of studies was insufficient and the sample size was small, leading to the failure to distinguish different types of cancer and to conduct subgroup analysis before and after treatment.

In conclusion, cancer promotes subclinical atherosclerosis, destroys functional and structural markers of blood vessels, and leads to higher IMT and PWV. The measurement of PWV and IMT values can be easily and quickly obtained by ultrasonic technology. Increased attention, early assessment, and timely prevention of atherosclerosis progression can reduce the incidence of cardiovascular accidents in cancer survivors.

## Data Availability Statement

The original contributions presented in the study are included in the article/Supplementary Material, further inquiries can be directed to the corresponding authors.

## Author Contributions

YD and DS contributed to the conception or design of the work. WZ, YD, and ZL contributed to the acquisition, analysis, or interpretation of data for the work. YD drafted the manuscript. DS, LC, and ZL critically revised the manuscript. All authors gave final approval and agree to be accountable for all aspects of work ensuring integrity and accuracy.

## Funding

This article was funded by the Jiangxi Provincial Department of Science and Technology key research and development program general project (20203BBGL73196) and the Natural Science Foundation of Jiangxi Province (20212BAB206040).

## Conflict of Interest

The authors declare that the research was conducted in the absence of any commercial or financial relationships that could be construed as a potential conflict of interest.

## Publisher's Note

All claims expressed in this article are solely those of the authors and do not necessarily represent those of their affiliated organizations, or those of the publisher, the editors and the reviewers. Any product that may be evaluated in this article, or claim that may be made by its manufacturer, is not guaranteed or endorsed by the publisher.
