# Supplementary material for: The Relationship Between Cancer and Functional and Structural Markers of Subclinical Atherosclerosis: A Systematic Review and Meta-Analysis
Source: Front Cardiovasc Med. 2022 May 4;9:849538. doi: 10.3389/fcvm.2022.849538 (PMC9115552; doi:10.3389/fcvm.2022.849538)
Supplement: Supplementary file 1 [file Data_Sheet_1.doc]

| Supplementary Material  **Table S1 Characteristics of literatures included in the meta-analysis concerning IMT.**   | author | year | study design | country | region | site measured | sample size | | mean age (y) | | male proportion (%) | | IMT (mm) | | | --- | --- | --- | --- | --- | --- | --- | --- | --- | --- | --- | --- | --- | --- | |  |  |  |  |  |  | **case** | **control** | **case** | **control** | **case** | **control** | **case** | **control** | | Dengel | 2019 | CS | USA | North America | BCCA | 108 | 83 | 26.4 | 22.2 | 61.0 | 54.0 | 0.5 | 0.5 | | Zaletel | 2018 | CS | Slovenia | Europe | BCCA | 43 | 52 | 40.2 | 42.7 | 30.0 | 23.0 | 0.5 | 0.5 | | Giordano | 2017 | CS | Italy | Europe | BCCA | 52 | 34 | 9.5 | 10.5 | 36.5 | 50.0 | 0.5 | 0.5 | | Sadurska | 2016 | CS | Poland | Europe | BCCA | 158 | 66 | 16.5 | 16.6 | 47.5 | 50.0 | 0.6 | 0.5 | | Okur | 2016 | CS | Turkey | Asia | BCCA | 50 | 30 | 13.5 | 12.0 | 70.0 | 60.0 | 0.5 | 0.5 | | Koelwyn | 2016 | CS | Canada | North America | BCCA | 30 | 30 | 61.0 | 62.0 | 0.0 | 0.0 | 0.7 | 0.7 | | Gujral | 2016 | CS | UK | Europe | BCCA,BIF | 50 | 50 | 58.0 | 58.0 | 68.0 | 68.0 | 0.8 | 0.7 | | Dengel | 2014 | CS | USA | North America | BCCA | 319 | 208 | 14.6 | 13.6 | 54.0 | 54.0 | 0.4 | 0.4 | | Brouwer | 2013 | CS | Netherland | Europe | BCCA,BIF,ICA | 277 | 130 | 28.0 | 26.0 | 56.0 | 52.0 | 0.6 | 0.6 | | Gianicolo | 2010 | CS | Italy | Europe | BCCA | 19 | 19 | 47.8 | 47.8 | 53.0 | 53.0 | 0.6 | 0.6 | | Chrisoulidou | 2010 | CS | Greece | Europe | CCA,BIF | 16 | 14 | 45.3 | 50.6 | 0.0 | 0.0 | 0.7 | 0.8 | | Meeske | 2009 | CS | USA | North America | RCCA | 30 | 30 | 27.5 | * | 63.0 | 60.0 | 0.5 | 0.4 | | Vaughn | 2008 | CS | USA | North America | RCCA | 24 | 15 | 41.4 | 44.7 | 100.0 | 100.0 | 0.8 | 0.8 | | Nastri | 2008 | CS | Brasil | South America | BCCA,BIF,ICA | 69 | 34 | 61.9 | 60.2 | 0.0 | 0.0 | 0.7 | 0.7 | | Simon | 2002 | CS | France | Europe | BCCA | 67 | 37 | 61.0 | 62.0 | 0.0 | 0.0 | 0.6 | 0.7 | | Feehs | 1991 | CS | USA | North America | BCCA,BIF,ICA | 29 | 9 | 62.6 | 58.7 | 68.0 | 67.0 | 1.3 | 0.9 | | Heikens | 2000 | CS | Netherland | Europe | BCCA,BIF,ICA | 26 | 29 | 25.8 | 27.7 | 46.0 | 53.0 | 0.6 | 0.6 | | Marlatt | 2019 | CS | USA | North America | BCCA | 13 | 10 | 26.6 | 26.9 | 42.9 | 61.5 | 0.6 | 0.6 | | Vassilakopoulou | 2010 | CS | Greece | Europe | BA | 27 | 10 | 61.0 | 55.0 | 0.0 | 0.0 | 0.6 | 0.5 | | Struder | 2020 | CS | Germany | Europe | BCCA | 117 | 20 | 62.3 | 61.5 | 82.9 | 75.0 | 0.7 | 0.6 | | Ye | 2012 | CS | USA | North America | ICA | 91 | 29 | 38.3 | 38.1 | 72.5 | 72.4 | 2.1 | 1.1 | | Huang | 2013 | CS | Chain | Asia | BCCA | 105 | 25 | 52.4 | 50.7 | 31.4 | 36.0 | 1.0 | 0.6 | | Yuan | 2017 | CS | Chain | Asia | BCCA | 139 | 76 | 56.1 | 42.8 | 47.5 | 51.3 | 0.7 | 0.5 | | Siviero-Miachon | 2015 | CS | Brasil | South America | BCCA | 55 | 24 | 18.6 | 19.8 | 43.6 | 41.7 | 0.6 | 0.6 | | Shariat | 2008 | CS | Malaysia | Asia | BCCA | 13 | 13 | 47.0 | 47.0 | 84.6 | 84.6 | 0.7 | 0.5 |   IMT, intima-media thickness; CS, cross-sectional study; BCCA, bilateral common carotid artery; BIF, carotid bifurcation; ICA, internal carotid artery; LCCA, left common carotid artery; RCCA, right common carotid artery; BA, brachial artery;*, not reported.  **Table S2 Characteristics of literatures included in the meta-analysis concerning PWV.**   | author | year | study design | country | region | site measured | sample size | | mean age (y) | | male proportion (%) | | PWV (m/s) | | | --- | --- | --- | --- | --- | --- | --- | --- | --- | --- | --- | --- | --- | --- | |  |  |  |  |  |  | **case** | **control** | **case** | **control** | **case** | **control** | **case** | **control** | | Zhang | 2017 | CS | Chain | Asia | CCA | 67 | 30 | 48.3 | 47.0 | 76.1 | 76.7 | 9.2 | 7.8 | | Zaletel | 2018 | CS | Slovenia | Europe | CCA | 43 | 52 | 40.2 | 42.7 | 30.0 | 23.0 | 6.3 | 5.7 | | Marlatt | 2019 | CS | USA | North America | CA-FA | 9 | 5 | 26.6 | 26.9 | 42.9 | 61.5 | 8.4 | 7.2 | | Kim | 2019 | CS | Korea | Asia | BA-AA | 49 | 17 | 62.0 | 66.0 | 65.3 | 94.1 | 15.5 | 16.7 | | Yersal | 2018 | CS | Turkey | Asia | BA-AA | 45 | 30 | 53.1 | 50.3 | 0.0 | 0.0 | 7.3 | 5.8 | | Krystal | 2015 | CS | USA | North America | CA-FA | 68 | 51 | 17.3 | 18.5 | 52.9 | 37.3 | 5.7 | 5.7 | | Dockery | 2000 | CS | UK | Europe | CA-FA | 12 | 12 | 70.6 | 70.1 | 100.0 | 100.0 | 14.2 | 11.8 | | Herceg-Cavrak | 2011 | CS | Croatia | Europe | CA-FA | 53 | 45 | 13.6 | 12.2 | 64.2 | 55.6 | 6.2 | 5.4 | | Sekijima | 2011 | CS | Japan | Asia | BA-AA | 14 | 12 | 56.9 | 55.3 | 0.0 | 0.0 | 15.9 | 16.7 | | Lim | 2010 | CS | Korea | Asia | BA-AA | 49 | 200 | 53.9 | 50.1 | 79.6 | 59.5 | 14.4 | 13.2 | | Chaosuwannakit | 2011 | CS | USA | North America | CA-FA | 13 | 13 | 52.0 | 53.0 | 30.0 | 62.0 | 4.5 | 4.6 | | Vrtovec | 2017 | CS | Slovenia | Europe | CCA | 40 | 42 | 57.1 | 58.2 | 35.0 | 38.1 | 6.2 | 6.5 | | Yuan | 2017 | CS | Chain | Asia | CCA | 139 | 76 | 56.1 | 42.8 | 47.5 | 51.3 | 8.8 | 6.3 | | Stelwagen | 2020 | CS | Netherland | Europe | CA-FA | 127 | 70 | 57.0 | 57.0 | 100.0 | 100.0 | 8.1 | 7.6 |   PWV, pulse wave velocity; CS, cross-sectional study; CA-FA, carotid artery to femoral artery; BA-AA, brachial artery to ankle artery; CCA, common carotid artery;  **Table S3 Characteristics of literatures included in the meta-analysis concerning FMD.**   | author | year | study design | country | region | site measured | sample size | | mean age (y) | | male proportion (%) | | FMD (%) | | | --- | --- | --- | --- | --- | --- | --- | --- | --- | --- | --- | --- | --- | --- | |  |  |  |  |  |  | **case** | **control** | **case** | **control** | **case** | **control** | **case** | **control** | | Giordano | 2017 | CS | Italy | Europe | BA | 52 | 34 | 9.5 | 10.5 | 36.5 | 50.0 | 9.8 | 14.6 | | Koelwyn | 2016 | CS | Canada | North America | BA | 30 | 30 | 61.0 | 62.0 | 0.0 | 0.0 | 5.1 | 5.2 | | Vatanen | 2015 | CS | Finland | Europe | BA | 19 | 20 | 22.7 | 22.4 | 42.0 | 45.0 | 10.2 | 9.6 | | Dengel | 2014 | CS | USA | North America | BA | 319 | 208 | 14.6 | 13.6 | 54.0 | 54.0 | 7.6 | 8.2 | | Brouwer | 2013 | CS | Netherland | Europe | BA | 277 | 130 | 28.0 | 26.0 | 56.0 | 52.0 | 5.5 | 5.9 | | Vaughn | 2008 | CS | USA | North America | BA | 24 | 15 | 41.4 | 44.7 | 100.0 | 100.0 | 5.6 | 8.8 | | Nastri | 2008 | CS | Brasil | South America | BA | 69 | 34 | 61.9 | 60.2 | 0.0 | 0.0 | 5.2 | 4.6 | | Sutterfield | 2018 | CS | USA | North America | BA | 7 | 7 | 55.0 | 54.0 | 14.0 | 14.0 | 2.2 | 6.6 | | Jenei | 2013 | CS | Hungary | Europe | BA | 96 | 72 | 14.9 | 13.7 | 59.0 | 58.0 | 8.0 | 13.1 | | Gilbert | 2013 | CS | UK | Europe | BA | 20 | 20 | 69.0 | 69.0 | 100.0 | 100.0 | 3.9 | 5.9 | | Tesarova | 2013 | CS | Czech Republic | Europe | BA | 50 | 20 | 38.5 | 38.5 | 0.0 | 0.0 | 6.2 | 7.1 | | Stamatelopoulos | 2004 | CS | Greece | Europe | BA | 14 | 13 | 62.7 | 58.3 | 0.0 | 0.0 | 4.6 | 3.7 | | Marlatt | 2019 | CS | USA | North America | BA | 12 | 10 | 26.6 | 26.9 | 42.9 | 61.5 | 6.7 | 6.7 | | Jones | 2013 | CS | USA | North America | BA | 10 | 10 | 51.0 | 46.0 | 0.0 | 0.0 | 5.7 | 5.2 | | Vassilakopoulou | 2010 | CS | Greece | Europe | BA | 27 | 10 | 61.0 | 55.0 | 0.0 | 0.0 | 5.6 | 5.6 | | Dardano | 2006 | CS | Italy | Europe | BA | 24 | 20 | 40.5 | 41.3 | 12.5 | 10.0 | 8.9 | 9.2 | | Ederer | 2016 | CS | Hungary | Europe | BA | 8 | 9 | 52.4 | 53.3 | 13.0 | 22.0 | 10.2 | 5.8 | | Beckman | 2001 | CS | USA | North America | BA | 16 | 10 | 58.0 | 58.0 | 0.0 | 0.0 | 3.8 | 2.5 |   FMD, flow mediated vasodilation; CS, cross-sectional study; BA, brachial artery;  **Table S4 The related covariates of literatures included in the meta-analysis.**   | **Author** | **Year** | **BMI (kg/m²)** | | **SBP (mmHg)** | | **DBP (mmHg)** | | **GLU (mmol/L)** | | **TC (mmol/L)** | | **TG (mmol/L)** | | **HDL-C (mmol/L)** | | **LDL-C (mmol/L)** | |  | | --- | --- | --- | --- | --- | --- | --- | --- | --- | --- | --- | --- | --- | --- | --- | --- | --- | --- | --- | |  |  | **case** | **control** | **case** | **control** | **case** | **control** | **case** | **control** | **case** | **control** | **case** | **control** | **case** | **control** | **case** | **control** |  | | **IMT** |  |  |  |  |  |  |  |  |  |  |  |  |  |  |  |  |  |  | | Dengel | 2019 | 62.3 | 23.9 | 116.2 | 116.1 | 66.7 | 61.8 | * | * | * | * | * | * | * | * | * | * |  | | Zaletel | 2018 | 24.9 | 25.4 | * | * | * | * | * | * | 5.6 | 5.7 | * | * | * | * | * | * |  | | Giordano | 2017 | 20.9 | 20.8 | 105.5 | 95.5 | 66.0 | 71.1 | * | * | 3.9 | 3.3 | 0.8 | 0.5 | 1.3 | 1.5 | 2.2 | 1.6 |  | | Sadurska | 2016 | 20.8 | 20.9 | 117.7 | 117.9 | 69.8 | 67.4 | * | * | 4.2 | 4.3 | 1.0 | 0.9 | 1.4 | 1.5 | 2.3 | 2.4 |  | | Okur | 2016 | * | * | * | * | * | * | * | * | * | * | * | * | * | * | * | * |  | | Koelwyn | 2016 | 25.3 | 25.4 | 128.0 | 137.0 | 78.0 | 83.0 | * | * | * | * | * | * | * | * | * | * |  | | Gujral | 2016 | 26.5 | 26.5 | * | * | * | * | * | * | 5.1 | 5.1 | * | * | 1.4 | 1.4 | 3.3 | 3.3 |  | | Dengel | 2014 | 22.4 | 21.8 | 110.9 | 110.5 | 58.3 | 57.5 | * | * | * | * | * | * | * | * | * | * |  | | Brouwer | 2013 | 22.8 | 23.7 | 144.0 | 115.0 | 79.0 | 77.0 | 4.7 | 4.6 | 4.7 | 4.5 | 1.0 | 1.0 | 1.3 | 1.4 | 2.9 | 2.7 |  | | Gianicolo | 2010 | * | * | * | * | * | * | * | * | * | * | * | * | * | * | * | * |  | | Chrisoulidou | 2010 | 26.8 | 28.8 | * | * | * | * | * | * | 4.7 | 4.5 | 0.9 | 1.4 | 1.4 | 1.3 | 2.3 | 2.5 |  | | Meeske | 2009 | 22.3 | 25.1 | 112.1 | 117.6 | 68.0 | 73.6 | 5.0 | 5.1 | 4.5 | 4.3 | 1.1 | 1.1 | 1.4 | 1.4 | 2.6 | 2.3 |  | | Vaughn | 2008 | 27.7 | 29.1 | 123.8 | 118.2 | 80.3 | 75.7 | * | * | * | * | 1.5 | 1.8 | 1.4 | 1.3 | * | * |  | | Nastri | 2008 | 25.2 | 25.6 | 134.7 | 133.4 | 81.9 | 80.4 | 5.0 | 5.0 | 6.1 | 6.3 | 1.4 | 1.4 | 1.3 | 1.3 | * | * |  | | Simon | 2002 | 26.0 | 26.0 | 121.0 | 122.0 | 69.0 | 68.0 | 5.5 | 5.5 | 5.8 | 6.0 | * | * | * | * | * | * |  | | Feehs | 1991 | * | * | 138.6 | 128.3 | * | * | * | * | * | * | * | * | * | * | * | * |  | | Heikens | 2000 | 23.6 | 25.4 | 121.0 | 110.0 | 77.0 | 75.0 | * | * | 4.9 | 4.5 | 1.1 | 0.7 | 1.1 | 1.4 | 3.3 | 2.8 |  | | Marlatt | 2019 | 31.8 | 30.1 | 119 | 129.0 | 68.0 | 72.0 | 4.7 | 4.1 | 4.6 | 4.4 | 1.4 | 1.2 | 1.2 | 1.3 | 2.7 | 2.7 |  | | Vassilakopoulou | 2010 | * | * | 133.6 | 134.7 | 76.8 | 79.0 | * | * | 5.8 | 5.4 | 1.0 | 1.7 | 1.5 | 1.4 | 5.9 | 3.2 |  | | Struder | 2020 | 24.8 | 26.6 | * | * | * | * | * | * | * | * | * | * | * | * | * | * |  | | Ye | 2012 | * | * | * | * | * | * | * | * | * | * | * | * | * | * | * | * |  | | Huang | 2013 | 23.8 | 22.6 | * | * | * | * | 5.1 | 4.8 | 5.2 | 4.8 | 1.3 | 1.0 | 1.6 | 1.8 | 3.1 | 2.6 |  | | Yuan | 2017 | * | * | 124.1 | 116.2 | 77.7 | 74.1 | * | * | * | * | * | * | * | * | * | * |  | | Siviero-Miachon | 2015 | * | * | 110.2 | 110.6 | 70.5 | 66.4 | * | * | * | * | 1.0 | 1.2 | 1.2 | 1.2 | 2.3 | 2.5 |  | | Shariat | 2008 | * | * | * | * | * | * | * | * | * | * | * | * | * | * | * | * |  | | **PWV** |  |  |  |  |  |  |  |  |  |  |  |  |  |  |  |  |  |  | | Zhang | 2017 | 22.9 | 22.4 | 122.0 | 120.0 | 74.0 | 73.0 | 4.7 | 4.3 | 3.8 | 4.0 | 1.6 | 1.3 | 1.4 | 1.4 | 1.0 | 1.0 |  | | Zaletel | 2018 | 24.9 | 25.4 | * | * | * | * | * | * | 5.6 | 5.7 | * | * | * | * | * | * |  | | Marlatt | 2019 | 31.8 | 30.1 | 119 | 129.0 | 68.0 | 72.0 | 4.7 | 4.1 | 4.6 | 4.4 | 1.4 | 1.2 | 1.2 | 1.3 | 2.7 | 2.7 |  | | Kim | 2019 | 25.5 | 24.7 | 125.0 | 126.0 | 82.0 | 80.0 | * | * | 4.6 | 4.1 | 1.2 | 1.2 | 1.2 | 1.3 | 2.9 | 2.5 |  | | Yersal | 2018 | 30.0 | 28.2 | 123.3 | 116.7 | 83.0 | 73.4 | * | * | 5.7 | 5.1 | 1.4 | 1.3 | 1.3 | 1.3 | 3.7 | 3.1 |  | | Krystal | 2015 | 23.4 | 23.7 | 114.3 | 114.0 | 72.2 | 69.4 | * | * | * | * | * | * | * | * | * | * |  | | Dockery | 2000 | 25.0 | 25.6 | 146.0 | 137.2 | * | * | * | * | 5.3 | 5.8 | 1.9 | 2.1 | 1.3 | 1.1 | 3.3 | 3.7 |  | | Herceg-Cavrak | 2011 | 20.2 | 19.0 | 109.8 | 114.4 | 61.1 | 63.0 | * | * | * | * | * | * | * | * | * | * |  | | Sekijima | 2011 | 22.1 | 21.8 | 125.2 | 133.7 | 75.0 | 79.4 | 9.4 | 7.3 | 5.3 | 6.0 | 1.3 | 1.4 | 1.4 | 1.5 | 2.9 | 3.7 |  | | Lim | 2010 | 25.3 | 24.1 | 131.1 | 125.4 | 74.1 | 74.2 | 3.3 | 3.7 | 5.0 | 5.2 | 1.7 | 1.7 | 1.4 | 1.5 | 3.5 | 3.3 |  | | Chaosuwannakit | 2011 | 27.6 | 28.0 | 123.0 | 120.6 | 74.4 | 80.2 | * | * | * | * | * | * | * | * | * | * |  | | Vrtovec | 2017 | 25.4 | 27.0 | 139.0 | 136.0 | 80.0 | 80.0 | 5.4 | 5.1 | 5.0 | 5.2 | 1.8 | 1.7 | 1.4 | 1.6 | 2.7 | 2.9 |  | | Yuan | 2017 | * | * | 124.1 | 116.2 | 77.7 | 74.1 | * | * | * | * | * | * | * | * | * | * |  | | Stelwagen | 2020 | 26.5 | 25.6 | 133.0 | 133.0 | 87.0 | 87.0 | 5.8 | 5.8 | 5.4 | 5.5 | * | * | * | * | * | * |  | | **FMD** |  |  |  |  |  |  |  |  |  |  |  |  |  |  |  |  |  |  | | Giordano | 2017 | 20.9 | 20.8 | 105.5 | 95.5 | 66.0 | 71.1 | * | * | 3.9 | 3.3 | 0.8 | 0.5 | 1.3 | 1.5 | 2.2 | 1.6 |  | | Koelwyn | 2016 | 25.3 | 25.4 | 128.0 | 137.0 | 78.0 | 83.0 | * | * | * | * | * | * | * | * | * | * |  | | Vatanen | 2015 | 21.4 | 23.6 | 129.0 | 122.0 | 75.0 | 70.0 | 5.3 | 5.1 | 4.5 | 4.1 | 1.3 | 0.9 | 1.6 | 1.5 | 2.7 | 2.4 |  | | Dengel | 2014 | 22.4 | 21.8 | 110.9 | 110.5 | 58.3 | 57.5 | * | * | * | * | * | * | * | * | * | * |  | | Brouwer | 2013 | 22.8 | 23.7 | 144.0 | 115.0 | 79.0 | 77.0 | 4.7 | 4.6 | 4.7 | 4.5 | 1.0 | 1.0 | 1.3 | 1.4 | 2.9 | 2.7 |  | | Vaughn | 2008 | 27.7 | 29.1 | 123.8 | 118.2 | 80.3 | 75.7 | * | * | * | * | 1.5 | 1.8 | 1.4 | 1.3 | * | * |  | | Nastri | 2008 | 25.2 | 25.6 | 134.7 | 133.4 | 81.9 | 80.4 | 5.0 | 5.0 | 6.1 | 6.3 | 1.4 | 1.4 | 1.3 | 1.3 | * | * |  | | Sutterfield | 2018 | 29.7 | 26.9 | * | * | * | * | * | * | * | * | * | * | * | * | * | * |  | | Jenei | 2013 | 21.3 | 20.4 | 121.3 | 120.0 | 81.3 | 80.0 | 4.5 | 4.7 | * | * | 1.2 | 0.8 | 1.4 | 1.6 | 2.2 | 2.2 |  | | Gilbert | 2013 | 29.6 | 28.4 | 145.0 | 139.0 | 79.0 | 79.0 | * | * | 5.1 | 4.9 | 2.0 | 1.2 | 1.4 | 1.5 | 2.8 | 2.9 |  | | Tesarova | 2013 | * | * | * | * | * | * | * | * | * | * | * | * | * | * | * | * |  | | Stamatelopoulos | 2004 | 27.1 | 28.1 | 141.1 | 144.5 | 80.5 | 88.7 | * | * | 5.7 | 6.4 | 1.4 | 1.3 | 1.4 | 1.4 | 3.7 | 4.4 |  | | Marlatt | 2019 | 31.8 | 30.1 | 119 | 129.0 | 68.0 | 72.0 | 4.7 | 4.1 | 4.6 | 4.4 | 1.4 | 1.2 | 1.2 | 1.3 | 2.7 | 2.7 |  | | Jones | 2013 | 29.0 | 28.0 | * | * | * | * | * | * | * | * | * | * | * | * | * | * |  | | Vassilakopoulou | 2010 | * | * | 133.6 | 134.7 | 76.8 | 79.0 | * | * | 5.8 | 5.4 | 1.0 | 1.7 | 1.5 | 1.4 | 5.9 | 3.2 |  | | Dardano | 2006 | 24.0 | 24.2 | 114.7 | 112.4 | 73.8 | 71.1 | * | * | 4.8 | 4.6 | 1.0 | 1.1 | 1.3 | 1.3 | 2.9 | 2.8 |  | | Ederer | 2016 | 25.3 | 24.0 | 120.9 | 126.9 | 78.0 | 81.7 | * | * | * | * | * | * | * | * | * | * |  | | Beckman | 2001 | * | * | * | * | * | * | * | * | 5.3 | 5.5 | * | * | * | * | 3.1 | 3.0 |  | |
| --- | --- | --- | --- | --- | --- | --- | --- | --- | --- | --- | --- | --- | --- | --- | --- | --- | --- | --- | --- | --- | --- | --- | --- | --- | --- | --- | --- | --- | --- | --- | --- | --- | --- | --- | --- | --- | --- | --- | --- | --- | --- | --- | --- | --- | --- | --- | --- | --- | --- | --- | --- | --- | --- | --- | --- | --- | --- | --- | --- | --- | --- | --- | --- | --- | --- | --- | --- | --- | --- | --- | --- | --- | --- | --- | --- | --- | --- | --- | --- | --- | --- | --- | --- | --- | --- | --- | --- | --- | --- | --- | --- | --- | --- | --- | --- | --- | --- | --- | --- | --- | --- | --- | --- | --- | --- | --- | --- | --- | --- | --- | --- | --- | --- | --- | --- | --- | --- | --- | --- | --- | --- | --- | --- | --- | --- | --- | --- | --- | --- | --- | --- | --- | --- | --- | --- | --- | --- | --- | --- | --- | --- | --- | --- | --- | --- | --- | --- | --- | --- | --- | --- | --- | --- | --- | --- | --- | --- | --- | --- | --- | --- | --- | --- | --- | --- | --- | --- | --- | --- | --- | --- | --- | --- | --- | --- | --- | --- | --- | --- | --- | --- | --- | --- | --- | --- | --- | --- | --- | --- | --- | --- | --- | --- | --- | --- | --- | --- | --- | --- | --- | --- | --- | --- | --- | --- | --- | --- | --- | --- | --- | --- | --- | --- | --- | --- | --- | --- | --- | --- | --- | --- | --- | --- | --- | --- | --- | --- | --- | --- | --- | --- | --- | --- | --- | --- | --- | --- | --- | --- | --- | --- | --- | --- | --- | --- | --- | --- | --- | --- | --- | --- | --- | --- | --- | --- | --- | --- | --- | --- | --- | --- | --- | --- | --- | --- | --- | --- | --- | --- | --- | --- | --- | --- | --- | --- | --- | --- | --- | --- | --- | --- | --- | --- | --- | --- | --- | --- | --- | --- | --- | --- | --- | --- | --- | --- | --- | --- | --- | --- | --- | --- | --- | --- | --- | --- | --- | --- | --- | --- | --- | --- | --- | --- | --- | --- | --- | --- | --- | --- | --- | --- | --- | --- | --- | --- | --- | --- | --- | --- | --- | --- | --- | --- | --- | --- | --- | --- | --- | --- | --- | --- | --- | --- | --- | --- | --- | --- | --- | --- | --- | --- | --- | --- | --- | --- | --- | --- | --- | --- | --- | --- | --- | --- | --- | --- | --- | --- | --- | --- | --- | --- | --- | --- | --- | --- | --- | --- | --- | --- | --- | --- | --- | --- | --- | --- | --- | --- | --- | --- | --- | --- | --- | --- | --- | --- | --- | --- | --- | --- | --- | --- | --- | --- | --- | --- | --- | --- | --- | --- | --- | --- | --- | --- | --- | --- | --- | --- | --- | --- | --- | --- | --- | --- | --- | --- | --- | --- | --- | --- | --- | --- | --- | --- | --- | --- | --- | --- | --- | --- | --- | --- | --- | --- | --- | --- | --- | --- | --- | --- | --- | --- | --- | --- | --- | --- | --- | --- | --- | --- | --- | --- | --- | --- | --- | --- | --- | --- | --- | --- | --- | --- | --- | --- | --- | --- | --- | --- | --- | --- | --- | --- | --- | --- | --- | --- | --- | --- | --- | --- | --- | --- | --- | --- | --- | --- | --- | --- | --- | --- | --- | --- | --- | --- | --- | --- | --- | --- | --- | --- | --- | --- | --- | --- | --- | --- | --- | --- | --- | --- | --- | --- | --- | --- | --- | --- | --- | --- | --- | --- | --- | --- | --- | --- | --- | --- | --- | --- | --- | --- | --- | --- | --- | --- | --- | --- | --- | --- | --- | --- | --- | --- | --- | --- | --- | --- | --- | --- | --- | --- | --- | --- | --- | --- | --- | --- | --- | --- | --- | --- | --- | --- | --- | --- | --- | --- | --- | --- | --- | --- | --- | --- | --- | --- | --- | --- | --- | --- | --- | --- | --- | --- | --- | --- | --- | --- | --- | --- | --- | --- | --- | --- | --- | --- | --- | --- | --- | --- | --- | --- | --- | --- | --- | --- | --- | --- | --- | --- | --- | --- | --- | --- | --- | --- | --- | --- | --- | --- | --- | --- | --- | --- | --- | --- | --- | --- | --- | --- | --- | --- | --- | --- | --- | --- | --- | --- | --- | --- | --- | --- | --- | --- | --- | --- | --- | --- | --- | --- | --- | --- | --- | --- | --- | --- | --- | --- | --- | --- | --- | --- | --- | --- | --- | --- | --- | --- | --- | --- | --- | --- | --- | --- | --- | --- | --- | --- | --- | --- | --- | --- | --- | --- | --- | --- | --- | --- | --- | --- | --- | --- | --- | --- | --- | --- | --- | --- | --- | --- | --- | --- | --- | --- | --- | --- | --- | --- | --- | --- | --- | --- | --- | --- | --- | --- | --- | --- | --- | --- | --- | --- | --- | --- | --- | --- | --- | --- | --- | --- | --- | --- | --- | --- | --- | --- | --- | --- | --- | --- | --- | --- | --- | --- | --- | --- | --- | --- | --- | --- | --- | --- | --- | --- | --- | --- | --- | --- | --- | --- | --- | --- | --- | --- | --- | --- | --- | --- | --- | --- | --- | --- | --- | --- | --- | --- | --- | --- | --- | --- | --- | --- | --- | --- | --- | --- | --- | --- | --- | --- | --- | --- | --- | --- | --- | --- | --- | --- | --- | --- | --- | --- | --- | --- | --- | --- | --- | --- | --- | --- | --- | --- | --- | --- | --- | --- | --- | --- | --- | --- | --- | --- | --- | --- | --- | --- | --- | --- | --- | --- | --- | --- | --- | --- | --- | --- | --- | --- | --- | --- | --- | --- | --- | --- | --- | --- | --- | --- | --- | --- | --- | --- | --- | --- | --- | --- | --- | --- | --- | --- | --- | --- | --- | --- | --- | --- | --- | --- | --- | --- | --- | --- | --- | --- | --- | --- | --- | --- | --- | --- | --- | --- | --- | --- | --- | --- | --- | --- | --- | --- | --- | --- | --- | --- | --- | --- | --- | --- | --- | --- | --- | --- | --- | --- | --- | --- | --- | --- | --- | --- | --- | --- | --- | --- | --- | --- | --- | --- | --- | --- | --- | --- | --- | --- | --- | --- | --- | --- | --- | --- | --- | --- | --- | --- | --- | --- | --- | --- | --- | --- | --- | --- | --- | --- | --- | --- | --- | --- | --- | --- | --- | --- | --- | --- | --- | --- | --- | --- | --- | --- | --- | --- | --- | --- | --- | --- | --- | --- | --- | --- | --- | --- | --- | --- | --- | --- | --- | --- | --- | --- | --- | --- | --- | --- | --- | --- | --- | --- | --- | --- | --- | --- | --- | --- | --- | --- | --- | --- | --- | --- | --- | --- | --- | --- | --- | --- | --- | --- | --- | --- | --- | --- | --- | --- | --- | --- | --- | --- | --- | --- | --- | --- | --- | --- | --- | --- | --- | --- | --- | --- | --- | --- | --- | --- | --- | --- | --- | --- | --- | --- | --- | --- | --- | --- | --- | --- | --- | --- | --- | --- | --- | --- | --- | --- | --- | --- | --- | --- | --- | --- | --- | --- | --- | --- | --- | --- | --- | --- | --- | --- | --- | --- | --- | --- | --- | --- | --- | --- | --- | --- | --- | --- | --- | --- | --- | --- | --- | --- | --- | --- | --- | --- | --- | --- | --- | --- | --- | --- | --- | --- | --- | --- | --- | --- | --- | --- | --- | --- | --- | --- | --- | --- | --- | --- | --- | --- | --- | --- | --- | --- | --- | --- | --- | --- | --- | --- | --- | --- | --- | --- | --- | --- | --- | --- | --- | --- | --- | --- | --- | --- | --- | --- | --- | --- | --- | --- | --- | --- | --- | --- | --- | --- | --- | --- | --- | --- | --- | --- | --- | --- | --- | --- | --- | --- | --- | --- | --- | --- | --- | --- | --- | --- | --- | --- | --- | --- | --- | --- | --- | --- | --- | --- | --- | --- | --- | --- | --- | --- | --- | --- | --- | --- | --- | --- | --- | --- | --- | --- | --- | --- | --- | --- | --- | --- | --- | --- | --- | --- | --- | --- | --- | --- | --- | --- | --- | --- | --- | --- | --- | --- | --- | --- | --- | --- | --- | --- | --- | --- | --- | --- | --- | --- | --- | --- | --- | --- | --- | --- | --- | --- | --- | --- | --- | --- | --- | --- | --- | --- | --- | --- | --- | --- | --- | --- | --- | --- | --- | --- | --- | --- | --- | --- | --- | --- | --- | --- | --- | --- | --- | --- | --- | --- | --- | --- | --- | --- | --- | --- | --- | --- | --- | --- | --- | --- | --- | --- | --- | --- | --- | --- | --- | --- | --- | --- | --- | --- | --- | --- | --- | --- | --- | --- | --- | --- | --- | --- | --- | --- | --- | --- | --- | --- | --- | --- | --- | --- | --- | --- | --- | --- | --- | --- | --- | --- | --- | --- | --- | --- | --- | --- | --- | --- | --- | --- | --- | --- | --- | --- | --- | --- | --- | --- | --- | --- | --- | --- | --- | --- | --- | --- | --- | --- | --- | --- | --- | --- | --- | --- | --- | --- | --- | --- | --- | --- | --- | --- | --- | --- | --- | --- | --- | --- | --- | --- | --- | --- | --- | --- | --- | --- | --- | --- | --- | --- | --- | --- | --- | --- | --- | --- | --- | --- | --- | --- | --- | --- | --- | --- | --- | --- | --- | --- | --- | --- | --- | --- | --- | --- | --- | --- | --- | --- | --- | --- | --- | --- | --- | --- | --- | --- | --- | --- | --- | --- | --- | --- | --- | --- | --- | --- | --- | --- | --- | --- | --- | --- | --- | --- | --- | --- | --- | --- | --- | --- | --- | --- | --- | --- | --- | --- | --- | --- | --- | --- | --- | --- | --- | --- | --- | --- | --- | --- | --- | --- | --- | --- | --- | --- | --- | --- | --- | --- | --- | --- | --- | --- | --- | --- | --- | --- | --- | --- | --- | --- | --- | --- | --- | --- | --- | --- | --- | --- | --- | --- | --- | --- | --- | --- | --- | --- | --- | --- | --- | --- | --- | --- | --- | --- | --- | --- | --- | --- | --- | --- | --- | --- | --- | --- | --- | --- | --- | --- | --- | --- | --- | --- | --- | --- | --- | --- | --- | --- | --- | --- | --- | --- | --- | --- | --- | --- | --- | --- | --- | --- | --- | --- | --- | --- | --- | --- | --- | --- | --- | --- | --- | --- | --- | --- | --- | --- | --- | --- | --- | --- | --- | --- | --- | --- | --- | --- | --- | --- | --- | --- | --- | --- | --- | --- | --- | --- | --- | --- | --- | --- | --- | --- | --- | --- | --- | --- | --- | --- | --- | --- | --- | --- | --- | --- | --- | --- | --- | --- | --- | --- | --- | --- | --- | --- | --- | --- | --- | --- | --- | --- | --- | --- | --- | --- | --- | --- | --- | --- | --- | --- | --- | --- | --- | --- | --- | --- | --- | --- | --- | --- | --- | --- | --- | --- | --- | --- | --- | --- | --- | --- | --- | --- | --- | --- | --- | --- | --- | --- | --- | --- | --- | --- | --- | --- | --- | --- | --- | --- | --- | --- | --- | --- | --- | --- | --- | --- | --- | --- | --- | --- | --- | --- | --- | --- | --- | --- | --- | --- | --- | --- | --- | --- | --- | --- | --- | --- | --- | --- | --- | --- | --- | --- | --- | --- | --- | --- | --- | --- | --- | --- | --- | --- | --- | --- | --- | --- | --- | --- | --- | --- | --- | --- | --- | --- | --- | --- | --- | --- | --- | --- | --- | --- | --- | --- | --- | --- | --- | --- | --- | --- | --- | --- | --- | --- | --- | --- | --- | --- | --- | --- | --- | --- | --- | --- | --- | --- | --- | --- | --- | --- | --- | --- | --- | --- | --- | --- | --- | --- | --- | --- | --- | --- | --- | --- | --- | --- | --- | --- | --- | --- | --- | --- | --- | --- | --- | --- | --- | --- | --- | --- | --- | --- | --- | --- | --- | --- | --- | --- | --- | --- | --- | --- | --- | --- | --- | --- | --- | --- | --- | --- | --- | --- | --- | --- | --- | --- | --- | --- | --- | --- | --- | --- | --- | --- | --- | --- | --- | --- | --- | --- | --- | --- | --- | --- | --- | --- | --- | --- | --- | --- | --- | --- | --- | --- | --- | --- | --- | --- | --- | --- | --- | --- | --- | --- | --- | --- | --- | --- | --- | --- | --- | --- | --- | --- | --- | --- | --- | --- | --- | --- | --- | --- | --- | --- | --- | --- | --- | --- | --- | --- | --- | --- | --- | --- | --- | --- | --- | --- | --- | --- | --- | --- | --- | --- | --- | --- | --- | --- | --- | --- | --- | --- | --- | --- | --- | --- | --- | --- | --- | --- | --- | --- | --- | --- | --- | --- | --- | --- | --- | --- | --- | --- | --- | --- | --- | --- | --- | --- | --- | --- | --- | --- | --- | --- | --- | --- | --- | --- | --- | --- | --- | --- | --- | --- | --- | --- | --- | --- | --- | --- | --- | --- | --- | --- | --- | --- | --- | --- | --- | --- | --- | --- | --- | --- | --- | --- | --- | --- | --- | --- | --- | --- | --- | --- | --- | --- | --- | --- | --- | --- | --- | --- | --- | --- | --- | --- | --- | --- | --- | --- | --- | --- | --- | --- | --- | --- | --- | --- | --- | --- | --- | --- | --- | --- | --- | --- | --- | --- | --- | --- | --- | --- | --- | --- | --- | --- | --- | --- | --- | --- | --- | --- | --- | --- | --- | --- | --- | --- | --- | --- | --- | --- | --- | --- | --- | --- | --- | --- | --- | --- | --- | --- | --- | --- | --- | --- | --- | --- | --- | --- | --- | --- | --- | --- | --- | --- | --- | --- | --- |

| **Author** | **Year** | **Smoker** | | **HP** | | **DM** | | **dyslipidemia** | |
| --- | --- | --- | --- | --- | --- | --- | --- | --- | --- |
|  |  | **(%)** | | **(%)** | | **(%)** | | **(%)** | |
|  |  | **case** | **control** | **case** | **control** | **case** | **control** | **case** | **control** |
| IMT |  |  |  |  |  |  |  |  |  |
| Dengel | 2019 | * | * | * | * | * | * | * | * |
| Zaletel | 2018 | 30.4 | 30.8 | 26.1 | 15.4 | 0.0 | 0.0 | * | * |
| Giordano | 2017 | * | * | * | * | * | * | * | * |
| Sadurska | 2016 | 5.7 | 12.1 | * | * | * | * | 25.9 | 22.7 |
| Okur | 2016 | * | * | * | * | * | * | * | * |
| Koelwyn | 2016 | 37.0 | 27.0 | 17.0 | 17.0 | * | * | * | * |
| Gujral | 2016 | 54.0 | 54.0 | 28.0 | 28.0 | 8.0 | 8.0 | 22.0 | 22.0 |
| Dengel | 2014 | * | * | * | * | * | * | * | * |
| Brouwer | 2013 | 25.0 | 33.0 | * | * | * | * | * | * |
| Gianicolo | 2010 | 15.8 | 15.8 | 21.0 | 26.3 | 5.3 | 15.8 | 30.5 | 26.3 |
| Chrisoulidou | 2010 | * | * | * | * | * | * | * | * |
| Meeske | 2009 | 73.0 | 67.0 | * | * | * | * | * | * |
| Vaughn | 2008 | * | * | * | * | * | * | * | * |
| Nastri | 2008 | * | * | * | * | * | * | * | * |
| Simon | 2002 | 9.0 | 19.0 | 18.0 | 22.0 | 0.0 | 5.0 | 12.0 | 19.0 |
| Feehs | 1991 | * | * | * | * | * | * | * | * |
| Heikens | 2000 | * | * | * | * | * | * | * | * |
| Marlatt | 2019 | * | * | * | * | * | * | * | * |
| Vassilakopoulou | 2010 | 14.8 | 10.0 | 18.5 | 0.0 | 14.8 | 0.0 | * | * |
| Struder | 2020 | 98.3 | 100.0 | 41.9 | 20.0 | 10.3 | 0.0 | 12.8 | 0.0 |
| Ye | 2012 | * | * | * | * | * | * | * | * |
| Huang | 2013 | * | * | * | * | * | * | * | * |
| Yuan | 2017 | * | * | * | * | * | * | * | * |
| Siviero-Miachon | 2015 | * | * | * | * | * | * | * | * |
| Shariat | 2008 | * | * | * | * | * | * | * | * |
| **PWV** |  |  |  |  |  |  |  |  |  |
| Zhang | 2017 | * | * | * | * | * | * | * | * |
| Zaletel | 2018 | 30.4 | 30.8 | 26.1 | 15.4 | 0.0 | 0.0 | * | * |
| Marlatt | 2019 | * | * | * | * | * | * | * | * |
| Kim | 2019 | 55.1 | 82.4 | 77.8 | 47.1 | 28.6 | 52.9 | 25.0 | 33.3 |
| Yersal | 2018 | 6.7 | 10.0 | 13.3 | 10.0 | 13.3 | 13.3 | * | * |
| Krystal | 2015 | * | * | * | * | * | * | * | * |
| Dockery | 2000 | 50.0 | 33.3 | * | * | * | * | * | * |
| Herceg-Cavrak | 2011 | * | * | * | * | * | * | * | * |
| Sekijima | 2011 | * | * | 21.4 | 8.3 | 0.0 | 0.0 | 0.0 | 8.3 |
| Lim | 2010 | * | * | * | * | * | * | * | * |
| Chaosuwannakit | 2011 | 0.0 | 0.0 | 33.0 | 15.0 | 13.0 | 8.0 | 23.0 | 23.0 |
| Vrtovec | 2017 | 40.0 | 26.2 | * | * | 7.5 | 0.0 | * | * |
| Yuan | 2017 | * | * | * | * | * | * | * | * |
| Stelwagen | 2020 | 16.0 | 10.0 | 69.0 | 52.0 | 2.0 | 3.0 | 20.0 | 7.0 |
| **FMD** |  |  |  |  |  |  |  |  |  |
| Giordano | 2017 | * | * | * | * | * | * | * | * |
| Koelwyn | 2016 | 37.0 | 27.0 | 17.0 | 17.0 | * | * | * | * |
| Vatanen | 2015 | 21.0 | 0.0 | * | * | * | * | * | * |
| Dengel | 2014 | * | * | * | * | * | * | * | * |
| Brouwer | 2013 | 25.0 | 33.0 | * | * | * | * | * | * |
| Vaughn | 2008 | * | * | * | * | * | * | * | * |
| Nastri | 2008 | * | * | * | * | * | * | * | * |
| Sutterfield | 2018 | * | * | * | * | * | * | * | * |
| Jenei | 2013 | * | * | * | * | * | * | * | * |
| Gilbert | 2013 | * | * | * | * | * | * | * | * |
| Tesarova | 2013 | * | * | * | * | * | * | * | * |
| Stamatelopoulos | 2004 | 21.4 | 46.2 | 50.0 | 38.5 | 7.1 | 15.4 | 28.6 | 46.2 |
| Marlatt | 2019 | * | * | * | * | * | * | * | * |
| Jones | 2013 | * | * | 10.0 | 40.0 | 40.0 | 20.0 | 10.0 | 30.0 |
| Vassilakopoulou | 2010 | 14.8 | 10.0 | 18.5 | 0.0 | 14.8 | 0.0 | * | * |
| Dardano | 2006 | * | * | * | * | * | * | * | * |
| Ederer | 2016 | * | * | * | * | * | * | * | * |
| Beckman | 2001 | * | * | * | * | * | * | * | * |

| **Author** | **Year** | **CRP（mg/L）** | | **WBC counts (X10^9^/L)** | | **PCT** | | **IL-6 (pg/ml)** | | **TNF-α(pg/ml)** | | **ET-1(pg/mL)** | | **t-PA（ng/mL）** | | **PAI-I antigen（ng/mL）** | | **sICAM-1（ng/mL）** | |
| --- | --- | --- | --- | --- | --- | --- | --- | --- | --- | --- | --- | --- | --- | --- | --- | --- | --- | --- | --- |
|  |  | **case** | **control** | **case** | **control** | **case** | **control** | **case** | **control** | **case** | **control** | **case** | **control** | **case** | **control** | **case** | **control** | **case** | **control** |
| **IMT** |  |  |  |  |  |  |  |  |  |  |  |  |  |  |  |  |  |  |  |
| Dengel | 2019 | * | * | * | * | * | * | * | * | * | * | * | * | * | * | * | * | * | * |
| Zaletel | 2018 | * | * | * | * | * | * | * | * | * | * | * | * | * | * | * | * | * | * |
| Giordano | 2017 | 5.2 | 1.9 | * | * | * | * | * | * | * | * | 2.1 | 2.1 | * | * | * | * | * | * |
| Sadurska | 2016 | * | * | * | * | * | * | * | * | * | * | * | * | * | * | * | * | * | * |
| Okur | 2016 | * | * | * | * | * | * | * | * | * | * | * | * | * | * | * | * | * | * |
| Koelwyn | 2016 | * | * | * | * | * | * | * | * | * | * | * | * | * | * | * | * | * | * |
| Gujral | 2016 | * | * | * | * | * | * | * | * | * | * | * | * | * | * | * | * | * | * |
| Dengel | 2014 | * | * | * | * | * | * | * | * | * | * | * | * | * | * | * | * | * | * |
| Brouwer | 2013 | 1.3 | 1.3 | * | * | * | * | * | * | * | * | * | * | 5.3 | 4.2 | 16.0 | 12.0 | * | * |
| Gianicolo | 2010 | * | * | * | * | * | * | * | * | * | * | * | * | * | * | * | * | * | * |
| Chrisoulidou | 2010 | 2.7 | ND | * | * | * | * | * | * | * | * | * | * | * | * | * | * | * | * |
| Meeske | 2009 | 17.9 | 19.6 | * | * | * | * | * | * | * | * | * | * | * | * | * | * | * | * |
| Vaughn | 2008 | 1.7 | 0.9 | * | * | * | * | * | * | * | * | * | * | * | * | * | * | 234.8 | 199.0 |
| Nastri | 2008 | * | * | * | * | * | * | * | * | * | * | * | * | * | * | * | * | * | * |
| Simon | 2002 | * | * | * | * | * | * | * | * | * | * | * | * | * | * | * | * | * | * |
| Feehs | 1991 | * | * | * | * | * | * | * | * | * | * | * | * | * | * | * | * | * | * |
| Heikens | 2000 | * | * | * | * | * | * | * | * | * | * | * | * | * | * | * | * | * | * |
| Marlatt | 2019 | * | * | * | * | * | * | * | * | * | * | * | * | * | * | * | * | * | * |
| Vassilakopoulou | 2010 | * | * | * | * | * | * | 3.4 | 3.9 | 1.9 | 1.8 | * | * | * | * | * | * | * | * |
| Struder | 2020 | * | * | * | * | * | * | * | * | * | * | * | * | * | * | * | * | * | * |
| Ye | 2012 | * | * | * | * | * | * | * | * | * | * | * | * | * | * | * | * | * | * |
| Huang | 2013 | 3.5 | 1.1 | 4.9 | 5.3 | * | * | * | * | * | * | * | * | * | * | * | * | * | * |
| Yuan | 2017 | * | * | * | * | * | * | * | * | * | * | * | * | * | * | * | * | * | * |
| Siviero-Miachon | 2015 | * | * | * | * | * | * | * | * | * | * | * | * | * | * | * | * | * | * |
| Shariat | 2008 | * | * | * | * | * | * | * | * | * | * | * | * | * | * | * | * | * | * |
| **PWV** |  |  |  |  |  |  |  |  |  |  |  |  |  |  |  |  |  |  |  |
| Zhang | 2017 | * | * | * | * | * | * | * | * | * | * | * | * | * | * | * | * | * | * |
| Zaletel | 2018 | * | * | * | * | * | * | * | * | * | * | * | * | * | * | * | * | * | * |
| Marlatt | 2019 | * | * | * | * | * | * | * | * | * | * | * | * | * | * | * | * | * | * |
| Kim | 2019 | * | * | * | * | * | * | * | * | * | * | * | * | * | * | * | * | * | * |
| Yersal | 2018 | * | * | * | * | 2.3 | 0.3 | * | * | * | * | * | * | * | * | * | * | * | * |
| Krystal | 2015 | * | * | * | * | * | * | * | * | * | * | * | * | * | * | * | * | * | * |
| Dockery | 2000 | * | * | * | * | * | * | * | * | * | * | * | * | * | * | * | * | * | * |
| Herceg-Cavrak | 2011 | * | * | * | * | * | * | * | * | * | * | * | * | * | * | * | * | * | * |
| Sekijima | 2011 | * | * | * | * | * | * | * | * | * | * | * | * | * | * | * | * | * | * |
| Lim | 2010 | * | * | * | * | * | * | * | * | * | * | * | * | * | * | * | * | * | * |
| Chaosuwannakit | 2011 | * | * | * | * | * | * | * | * | * | * | * | * | * | * | * | * | * | * |
| Vrtovec | 2017 | 5.0 | 5.4 | 7.6 | 7.0 | * | * | * | * | * | * | * | * | * | * | * | * | * | * |
| Yuan | 2017 | * | * | * | * | * | * | * | * | * | * | * | * | * | * | * | * | * | * |
| Stelwagen | 2020 | 1.4 | 1.0 | * | * | * | * | * | * | * | * | * | * | 14.0 | 12.0 | 31.0 | 3.0 | * | * |
| **FMD** |  |  |  |  |  |  |  |  |  |  |  |  |  |  |  |  |  |  |  |
| Giordano | 2017 | 5.2 | 1.9 | * | * | * | * | * | * | * | * | 2.1 | 2.1 | * | * | * | * | * | * |
| Koelwyn | 2016 | * | * | * | * | * | * | * | * | * | * | * | * | * | * | * | * | * | * |
| Vatanen | 2015 | 3.5 | 2.6 | * | * | * | * | * | * | * | * | * | * | * | * | * | * | * | * |
| Dengel | 2014 | * | * | * | * | * | * | * | * | * | * | * | * | * | * | * | * | * | * |
| Brouwer | 2013 | 1.3 | 1.3 | * | * | * | * | * | * | * | * | * | * | 5.3 | 4.2 | 16.0 | 12.0 | * | * |
| Vaughn | 2008 | 1.7 | 0.9 | * | * | * | * | * | * | * | * | * | * | * | * | * | * | 234.8 | 199.0 |
| Nastri | 2008 | * | * | * | * | * | * | * | * | * | * | * | * | * | * | * | * | * | * |
| Sutterfield | 2018 | * | * | * | * | * | * | * | * | * | * | * | * | * | * | * | * | * | * |
| Jenei | 2013 | 1.1 | 1.1 | * | * | * | * | * | * | * | * | * | * | * | * | * | * | * | * |
| Gilbert | 2013 | * | * | * | * | * | * | * | * | * | * | * | * | * | * | * | * | * | * |
| Tesarova | 2013 | * | * | * | * | * | * | * | * | * | * | * | * | * | * | * | * | * | * |
| Stamatelopoulos | 2004 | * | * | * | * | * | * | * | * | * | * | * | * | * | * | * | * | * | * |
| Marlatt | 2019 | * | * | * | * | * | * | * | * | * | * | * | * | * | * | * | * | * | * |
| Jones | 2013 | * | * | * | * | * | * | * | * | * | * | * | * | * | * | * | * | * | * |
| Vassilakopoulou | 2010 | * | * | * | * | * | * | 3.4 | 3.9 | 1.9 | 1.8 | * | * | * | * | * | * | * | * |
| Dardano | 2006 | 0.4 | 0.4 | * | * | * | * | 19.8 | 20.1 | 26.5 | 23.2 | * | * | * | * | * | * | * | * |
| Ederer | 2016 | * | * | * | * | * | * | * | * | * | * | * | * | * | * | * | * | * | * |
| Beckman | 2001 | * | * | * | * | * | * | * | * | * | * | * | * | * | * | * | * | * | * |

FMD indicates flow-mediated vasodilation; IMT, intima media thickness; PWV, pulse wave velocity; BMI, body mass index; SBP, systolic blood pressure; DBP, diastolic blood pressure; GLU, fasting blood glucose; TC, total cholesterol; TG, triglyceride; HDL, high density lipoprotein, LDL, low density lipoprotein; HP, hypertension; DM, diabetes mellitus;CRP, C-reactive protein; WBC:white blood cell; PCT, Procalcitonin; IL-6, interleukin-6; TNF-α, tumor necrosis factor α;ET-1, endothelin-1; t-PA, tissue-type plasminogen activator; PAI-I, Tissue type I plasminogen activator inhibitor;sICAM-1, intercellular adhesion molecule-1; *, not reported.

| **Table S5. WMDs (95% CI) of sensitivity analysis concerning IMT** | | |
| --- | --- | --- |
| Excluding literature one by one | year | SMD (95% CI) |
| Over all |  | 0.290(0.069, 0.511) |
| Dengel | 2019 | 0.295(0.061, 0.530) |
| Zaletel | 2018 | 0.302(0.073, 0.532) |
| Giordano | 2017 | 0.309(0.082, 0.537) |
| Sadurska | 2016 | 0.272(0.043, 0.501) |
| Okur | 2016 | 0.280(0.051, 0.508) |
| Koelwyn | 2016 | 0.318(0.093, 0.543) |
| Gujral | 2016 | 0.280(0.051, 0.510) |
| Dengel | 2014 | 0.305(0.065, 0.544) |
| Brouwer | 2013 | 0.305(0.068, 0.542) |
| Gianicolo | 2010 | 0.294(0.067, 0.521) |
| Chrisoulidou | 2010 | 0.327(0.106, 0.548) |
| Meeske | 2009 | 0.280(0.052, 0.508) |
| Vaughn | 2008 | 0.355(0.146, 0.563) |
| Nastri | 2008 | 0.305(0.076, 0.534) |
| Simon | 2002 | 0.322(0.099, 0.545) |
| Feehs | 1991 | 0.258(0.037, 0.480) |
| Heikens | 2000 | 0.301(0.073, 0.529) |
| Marlatt | 2019 | 0.293(0.067, 0.518) |
| Vassilakopoulou | 2010 | 0.284(0.058, 0.510) |
| Struder | 2020 | 0.270(0.044, 0.496) |
| Ye | 2012 | 0.244(0.030, 0.459) |
| Huang | 2013 | 0.267(0.041, 0.493) |
| Yuan | 2017 | 0.246(0.039, 0.453) |
| Siviero, Miachon | 2015 | 0.285(0.057, 0.514) |
| Shariat | 2008 | 0.249(0.031, 0.468) |

SMD, standardized mean difference; CI, confidence interval; IMT, intima-media thickness; The study was in bold if the result was reversed after removement.

| **Table S6. WMDs (95% CI) of sensitivity analysis concerning PWV** |
| --- |
| | Excluding literature one by one | year | SMD (95% CI) | | --- | --- | --- | | Over all |  | 0.392(0.136, 0.647) | | Zhang | 2017 | 0.385(0.108, 0.662) | | Zaletel | 2018 | 0.377(0.099, 0.654) | | Marlatt | 2019 | 0.388(0.124, 0.651) | | Kim | 2019 | 0.450(0.198, 0.701) | | Yersal | 2018 | 0.332(0.079, 0.585) | | Krystal | 2015 | 0.419(0.147, 0.690) | | Dockery | 2000 | 0.358(0.097, 0.619) | | Herceg, Cavrak | 2011 | 0.360(0.087, 0.632) | | Sekijima | 2011 | 0.430(0.170, 0.689) | | Lim | 2010 | 0.380(0.095, 0.666) | | Chaosuwannakit | 2011 | 0.420(0.157, 0.682) | | Vrtovec | 2017 | 0.447(0.195, 0.700) | | Yuan | 2017 | 0.330(0.090, 0.570) | | Stelwagen | 2020 | 0.406(0.124, 0.688) | |

SMD, standardized mean difference; CI, confidence interval; PWV, pulse wave velocity; The study was in bold if the result was reversed after removement.

| **Table S7 WMDs (95% CI) of sensitivity analysis concerning FMD** |
| --- |
| | Excluding literature one by one | year | SMD (95% CI) | | --- | --- | --- | | Over all |  | -0.192(-0.527, 0.144) | | Giordano | 2017 | -0.129(-0.469, 0.211) | | Koelwyn | 2016 | -0.201(-0.556, 0.154) | | Vatanen | 2015 | -0.209(-0.560, 0.142) | | Dengel | 2014 | -0.197(-0.601, 0.207) | | Brouwer | 2013 | -0.202(-0.593, 0.189) | | Vaughn | 2008 | -0.068(-0.378, 0.242) | | Nastri | 2008 | -0.225(-0.578, 0.129) | | Sutterfield | 2018 | -0.113(-0.437, 0.211) | | Jenei | 2013 | -0.126(-0.457, 0.205) | | Gilbert | 2013 | -0.163(-0.513, 0.187) | | Tesarova | 2013 | -0.201(-0.555, 0.154) | | Stamatelopoulos | 2004 | -0.214(-0.562, 0.134) | | Marlatt | 2019 | -0.202(-0.550, 0.146) | | Jones | 2013 | -0.216(-0.563, 0.130) | | Vassilakopoulou | 2010 | -0.203(-0.552, 0.147) | | Dardano | 2006 | -0.198(-0.551, 0.155) | | Ederer | 2016 | -0.275(-0.605, 0.056) | | Beckman | 2001 | -0.312(-0.625, 0.002) | |

SMD, standardized mean difference; CI, confidence interval; FMD, flow mediated vasodilation; The study was in bold if the result was reversed after removement.

**Table S8 The results of meta-regression.**

| Covariate | IMT | | | | Covariate | PWV | | | | Covariate | FMD | | | |
| --- | --- | --- | --- | --- | --- | --- | --- | --- | --- | --- | --- | --- | --- | --- |
|  | N | P | Coefficient | 95% CI |  | N | P | Coefficient | 95% CI |  | N | P | Coefficient | 95% CI |
| Sample measured | 25 | 0.299 | 0.296 | -0.280,0.872 | Sample measured | 14 | 0.712 | 0.112 | -0.533,0.756 | Sample measured | 18 | 0.951 | -0.048 | -1.690, 1.595 |
| ＜100 | 14 | 0.299 | -0.296 | -0.872, 0.280 | ＜100 | 10 | 0.712 | -0.112 | -0.756, 0.533 | ＜100 | 14 | 0.951 | 0.048 | -1.595, 1.690 |
| ≥100 | 11 | 0.299 | 0.296 | -0.280, 0.872 | ≥100 | 4 | 0.712 | 0.112 | -0.533, 0.756 | ≥100 | 4 | 0.951 | -0.048 | -1.690, 1.595 |
| I^2^= 86.18%% R^2^=1.64% | | | | | I^2^= 78.49% R^2^=-11.29% | | | | | I^2^= 88.83% R^2^=-10.04% | | | | |
| age | 25 | 0.394 | 0.254 | -0.351, 0.858 | age | 14 | 0.715 | -0.110 | -0.748, 0.529 | age | 18 | 0.249 | 0.751 | -0.579, 2.080 |
| ＜50 | 16 | 0.394 | -0.254 | -0.858, 0.351 | ＜50 | 5 | 0.715 | 0.110 | -0.529, 0.748 | ＜50 | 9 | 0.249 | -0.751 | -2.080, 0.579 |
| ≥50 | 9 | 0.394 | 0.254 | -0.351, 0.858 | ≥50 | 9 | 0.715 | -0.110 | -0.748, 0.529 | ≥50 | 9 | 0.249 | 0.751 | -0.579, 2.080 |
| I^2^= 85.40% R^2^=-1.75% | | | | | I^2^= 78.81% R^2^=-11.38% | | | | | I^2^= 87.79% R^2^=8.1% | | | | |
| Male ratio(%) | 25 | 0.366 | 0.259 | -0.322, 0.839 | Male ratio(%) | 14 | 0.829 | -0.062 | -0.676, 0.552 | Male ratio(%) | 18 | 0.165 | -0.976 | -2.398, 0.446 |
| ＜50 | 13 | 0.366 | -0.259 | -0.839, o.322 | ＜50 | 7 | 0.829 | 0.062 | -0.552, 0.676 | ＜50 | 13 | 0.165 | 0.976 | -0.446, 2.398 |
| ≥50 | 12 | 0.366 | 0.259 | -0.322, 0.839 | ≥50 | 7 | 0.829 | -0.062 | -0.676, 0.552 | ≥50 | 5 | 0.165 | -0.976 | -2.398, 0.446 |
| I^2^= 86.38% R^2^=-2.00% | | | | | I^2^= 77.05% R^2^=-10.13% | | | | | I^2^= 88.34% R^2^=12.73% | | | | |
| Region | 25 | 0.160 | -0.236 | -0.573, 0.100 | Region | 14 | 0.405 | -0.159 | -0.561, 0.243 | Region | 18 | 0.913 | -0.060 | -1.217, 1.096 |
| Asia | 4 | **0.020** | 0.860 | 0.151, 1.569 | Asia | 6 | 0.602 | 0.150 | -0.459, 0.759 | Europe | 10 | 0.759 | 0.209 | -1.206, 1.623 |
| Europe | 11 | 0.288 | -0.304 | -0.882, 0.274 | Europe | 5 | 0.852 | 0.056 | -0.581, 0.692 | North America | 7 | 0.622 | -0.342 | -1.785, 1.100 |
| North America | 8 | 0.641 | -0.145 | -0.780, 0.490 | North America | 3 | 0.358 | -0.342 | -1.121, 0.438 | South America | 1 | 0.716 | 0.517 | -2.438, 3.473 |
| South America | 2 | 0.811 | -0.125 | -1.194, 0.944 | I^2^= 74.19% R^2^=2.36% | | | | | I^2^= 88.24% R^2^=-10.09% | | | | |
| I^2^= 84.25% R^2^=5.76% | | | | | Site measured | 14 | 0.660 | -0.081 | -0.474, 0.311 |  |  |  |  |  |
| Site measured | 25 | 0.272 | 0.217 | -0.182, 0.616 | CCA | 4 | 0.713 | 0.113 | -0.540, 0.765 |  |  |  |  |  |
| CCA,BIF,ICA | 2 | 0.499 | -0.343 | -1.373, 0.688 | CA-FA | 6 | 1.000 | 0.000 | -0.629, 0.629 |  |  |  |  |  |
| CCA,BIF | 4 | 0.892 | -0.055 | -0.883, 0.773 | BA-AA | 4 | 0.705 | -0.121 | -0.801, 0.558 |  |  |  |  |  |
| CCA | 17 | 0.820 | -0.071 | -0.707, 0.565 | I^2^= 78.22% R^2^=-10.7% | | | | |  |  |  |  |  |
| other | 2 | 0.199 | 0.699 | -0.396, 1.794 |  |  |  |  |  |  |  |  |  |  |
| I^2^= 85.21% R^2^=2.63% | | | | |  |  |  |  |  |  |  |  |  |  |

FMD indicates flow-mediated vasodilation; IMT, intima media thickness; PWV, pulse wave velocity; CI, confidence interval; CCA, common carotid artery; BIF, carotid bifurcation; ICA, internal carotid artery; BA, brachial artery; CA-FA, carotid artery to femoral artery; BA-AA, brachial artery to ankle artery; The results were in bold, if *p* ＜ 0.05.
