# Supplementary material for: The Relationship Between Cancer and Functional and Structural Markers of Subclinical Atherosclerosis: A Systematic Review and Meta-Analysis
Source: Front Cardiovasc Med. 2022 May 4;9:849538. doi: 10.3389/fcvm.2022.849538 (PMC9115552; doi:10.3389/fcvm.2022.849538)
Supplement: Supplementary file 2 [file Data_Sheet_2.docx]

Supplementary Material

# Supplementary Tables

Table S1 Characteristics of literature included in the meta-analysis concerning IMT.

IMT, intima-media thickness; CS, cross-sectional study; BCCA, bilateral common carotid artery; BIF, carotid bifurcation; ICA, internal carotid artery; LCCA, left common carotid artery; RCCA, right common carotid artery; BA, brachial artery; *, not reported.

Table S2 Characteristics of literature included in the meta-analysis concerning PWV.

PWV, pulse wave velocity; CS, cross-sectional study; CA-FA, carotid artery to femoral artery; BA-AA, brachial artery to ankle artery; CCA, common carotid artery;

Table S3 Characteristics of literature included in the meta-analysis concerning FMD

FMD, flow mediated vasodilation; CS, cross-sectional study; BA, brachial artery;

Table S4 The related covariates of literature included in the meta-analysis.

FMD indicates flow-mediated vasodilation; IMT, intima media thickness; PWV, pulse wave velocity; BMI, body mass index; SBP, systolic blood pressure; DBP, diastolic blood pressure; GLU, fasting blood glucose; TC, total cholesterol; TG, triglyceride; HDL, high density lipoprotein, LDL, low density lipoprotein; HP, hypertension; DM, diabetes mellitus; CRP, C-reactive protein; WBC: white blood cell; PCT, Procalcitonin; IL-6, interleukin-6; TNF-α, tumor necrosis factor α;ET-1, endothelin-1; t-PA, tissue-type plasminogen activator; PAI-I, Tissue type I plasminogen activator inhibitor;sICAM-1, intercellular adhesion molecule-1; *, not reported.

Table S5. WMDs (95% CI) of sensitivity analysis concerning IMT.

SMD, standardized mean difference; CI, confidence interval; IMT, intima-media thickness; The study was in bold if the result was reversed after removement

Table S6. WMDs (95% CI) of sensitivity analysis concerning PWV.

SMD, standardized mean difference; CI, confidence interval; PWV, pulse wave velocity; The study was in bold if the result was reversed after removement.

Table S7 WMDs (95% CI) of sensitivity analysis concerning FMD

SMD, standardized mean difference; CI, confidence interval; FMD, flow-mediated vasodilation; The study was in bold if the result was reversed after removement.

Table S8 The results of meta-regression.

FMD indicates flow-mediated vasodilation; IMT, intima media thickness; PWV, pulse wave velocity; CI, confidence interval; CCA, common carotid artery; BIF, carotid bifurcation; ICA, internal carotid artery; BA, brachial artery; CA-FA, carotid artery to femoral artery; BA-AA, brachial artery to ankle artery; The results were in bold if p ＜ 0.05.
